# Eye care utilization pattern in South Africa: results from SANHANES-1

**DOI:** 10.1186/s12913-020-05621-8

**Published:** 2020-08-17

**Authors:** Kwadwo Owusu Akuffo, Ronel Sewpaul, Natisha Dukhi, Akosua Kesewah Asare, David Ben Kumah, Emmanuel Kofi Addo, Eldad Agyei-Manu, Priscilla Reddy

**Affiliations:** 1 Department of Optometry and Visual Science, College of Science, Kwame Nkrumah University of Science and Technology, Kumasi, Ghana; 2 Social Aspects of Public Health, Human Sciences Research Council, Cape Town, South Africa; 3 Department of Ophthalmology and Visual Sciences, Moran Eye Centre, University of Utah, Salt Lake City, Utah United States; 4 Department of Nutrition and Integrative Physiology, University of Utah, Salt Lake City, Utah United States; 5 Usher Institute for Population Health Sciences and Informatics, College of Medicine and Veterinary Medicine, University of Edinburgh, Edinburgh, United Kingdom; 6 Research Associate, Faculty of Health Sciences, Nelson Mandela University, Port Elizabeth, South Africa

**Keywords:** Utilisation, Eye care utilization, Barriers to eye care utilization, Eye care services, Access to healthcare, Social determinants of health and (primary and secondary) prevention

## Abstract

**Background:**

Eye examinations are recommended for all persons throughout life. However, there is disparity in the uptake of eye care services in different populations. Using data from a nationally representative population-based cross-sectional study (the South African National Health and Nutrition Examination Survey, [SANHANES-1]), this paper investigates the utilization of eye care services and its associated factors in South Africa.

**Methods:**

Participants aged 15 years and older who participated in interviews and clinical examination were enrolled in the SANHANES from 2011 to 2012. Eye care utilization was assessed from participants’ responses to whether they had their eyes examined by a medical professional and when they were last examined. Data were analysed using multiple logistic regression models employing a hierarchical approach to add predisposing (e.g. age, sex), enabling (e.g. health insurance) and need (e.g. hypertension) factors sequentially.

**Results:**

The study sampled 3320 participants, with 64.9% being females. 73.4% (95% CI [69.7–76.7]) of participants had never had an eye examination. After statistical adjustment, age groups (compared with 15–29 years: 30-44 years Odds Ratio [OR] = 1.76; 45-59 years OR = 2.13; 60-74 years OR = 2.74; ≥75 years OR = 3.22), ethnicity (compared with African descent: white OR = 4.71; mixed-race OR = 1.87; Indian OR = 7.67), high risk alcohol use (OR = 1.83), wealth index (compared with lowest quintile: third quintile OR = 1.75; fourth quintile OR = 2.23; fifth quintile OR = 2.49), health insurance (OR = 2.19), diabetes (OR = 1.75), high cholesterol (OR = 2.51), having assessed healthcare in the past 5 years (OR = 2.42), and self-reported vision problems (OR = 1.51) were significantly associated with eye care utilization.

**Conclusion:**

Almost three-quarters of South Africans sampled were not utilizing eye care services. It is imperative to strengthen current public health measures (including eye health promotion programs) to address the alarmingly low uptake of eye care services as well as the disparities in eye care utilization in South Africa.

## Background

Blindness and visual impairment are major health problems, particularly in low- and middle-income countries. The burden of visual impairment is not evenly distributed throughout the world; the least developed countries usually carry the greatest burden, with the majority of the global burden of visual impairment being found in India and Africa [1].

Health care utilization is often defined as the quality of health care services and the use of these services by individuals for disease prevention, promotion of health and wellbeing, as well as obtaining information on one’s health status and prognosis [2]. The utilization of eye care services reflects the effective coverage of healthcare service and is a marker of vision health system performance. Anderson’s model of healthcare classifies utilization into predisposing factors, need factors and enabling factors [3]. Predisposing factors reflect the tendency for people with certain characteristics to utilize health services, even though these attributes may not be directly responsible for health service utilization. Predisposing factors include age, sex, ethnicity, and lifestyle. Enabling factors are surrounding circumstances that affect an individual’s ability to effectively utilize health services. They include wealth, availability of health insurance, income and proximity to a health facility. Need factors refer to the presence of ill-health or the perception of threat.

A systematic review and meta-analysis of global causes of blindness by Flaxman et al. [4], revealed that, in 2015, the proportion of blindness attributable to glaucoma was highest in Southern Sub-Saharan Africa. Projections made into 2020 showed a decline in the prevalence of glaucoma and visual impairment; however, this projected decline was less marked in the sub-Saharan Africa region. This calls for conscientious efforts in improving eye care education and service delivery in sub-Saharan Africa. The World Health Survey of eye care utilization conducted in 70 countries, reported that the percentage of adults who had an eye exam in the last 1 year in low, lower-middle, upper-middle, and high-income countries was 10, 24, 22, and 37% respectively [5], showing a trend of increasing eye care utilization among countries with high-income status. Age, ethnicity, wealth, health insurance, diabetes, and self-reported visual problems were also found to be positively associated with eye care utilization. In Africa, availability, accessibility, and affordability have been found to be barriers to eye care utilization [5–8].

South Africa is an upper-middle-income country [9] with a population of about 58.78 million [10]. Eye care services are mainly provided by optometrists and ophthalmologists. However, there is a huge disparity between the care (services needed by the patients and that provided by healthcare facilities/personnel) [11]. Of note, several studies have shown that more than 70% of cataract patients who did not seek treatment attributed their inability to seek healthcare on financial commitment that is associated with treatment [12–14]. On the contrary, individuals with systemic diseases (such as diabetes, hypertension and high cholesterol) are more likely to utilize eye care services than those without systemic diseases [8].

Since the early 2000s, efforts have been made towards the promotion of eye health, including the introduction of provincial eyecare coordinators at the Directorate of Health Promotion (South Africa). However, majority of these infrastructures are woefully underfunded. In recent times, significant effort has been made by academic institutions and non-governmental organizations such as the International Centre for Eyecare Education (ICEE) in mobilizing community participation in eye health promotion and strategy development. In terms of governmental effort, the National Prevention of Blindness Program set out some guidelines in 2003 [15] intended to coordinate and provide support towards blindness prevention, improving access to primary eyecare and promoting the right of persons living with blindness. Although well-intended, these interventions tend to address curative eye health [16]. Eye health promotion requires a holistic approach that empowers both individuals and communities [17] to take action for their eye health as well as strengthening sectoral partnerships at the district and national level. A prospective study [18] which evaluated primary eyecare services in three South African districts revealed that introducing a health system strengthening (HSS) package strengthened primary eyecare through improvement in organizational care and clinical practice. Therefore, assessing the patterns of eye care utilization is essential for appraising current eye health promotion systems [6, 14].

The purpose of this study is to evaluate the pattern of eye care utilization among children and adults (aged 15 years or older) in South Africa using data from the South African National Health and Nutrition Examination Survey (SANHANES-1). This study presents a secondary analysis of data obtained from SANHANES-1 and will provide (for the first time) nationally representative data on the eye care-seeking patterns of South Africans.

## Methods

### Data and sample

This cross-sectional study used secondary analyses of data from the SANHANES-1, a population-based national survey conducted in 2011–2012 [19]. Participants’ data were obtained through interviews, clinical examinations and biomarker analyses. Additional details of SANHANES-1 methodology and laboratory procedures are reported in Shisana et al [19]. Criteria for inclusion included: 1) persons of all ages living in South Africa; 2) persons living in occupied households (HHs). Exclusion criteria included persons staying in educational institutions, old-age homes, hospitals, homeless people, and uniformed-service barracks.

Multi-stage disproportionate, stratified cluster sampling was used to select households within enumeration areas (EAs) stratified by province and locality type. A total of 10,000 households were selected. Within the occupied households, 27,580 individuals of all ages were eligible to be interviewed and agreed to participate; out of which 25,532 (92.6%) completed the interview. Of the latter number, 12025 (43.6%) individuals volunteered to undergo a medical examination. Data was analyzed on individuals aged 15 years or older who participated in the interview and the physical examination and who responded to the questions about eye care utilization.

### Ethical approval

Ethical approval for the study was obtained from the Research Ethics Committee (REC) of the South African Human Sciences Research Council (HSRC) (REC number: 6/16/11/11). Informed written consent/assent was obtained from all the survey participants. Parent/guardian consent was also obtained for all children aged 17 or younger. The study procedures adhered to the tenets of the Declaration of Helsinki.

### Measures

Eye care utilization was assessed from participants’ responses to whether they had their eyes examined by a medical professional and when they last had their eyes examined. A medical professional is an individual with the required medical qualifications and skills to undertake eye examination (such as an optometrist/ophthalmologist/ophthalmic nurse/medical doctor).

The individual factors associated with having had an eye examination by a medical professional were investigated using Andersen’s Behavioural Model [3]. According to this model, variables were categorized into predisposing, enabling, and need factors.

Predisposing factors included sex, age group, ethnicity, risky alcohol use and current tobacco smoking, which were all based on self-report. Risky alcohol use was assessed by the AUDIT-C, a 3-item alcohol screening tool indicating hazardous drinking or active alcohol use disorders [20]. High-risk alcohol use referred to hazardous drinking or active alcohol use disorders, as measured by a score of 4 or more for men and 3 or more for women, in the AUDIT-C 3-item alcohol use screening tool.

The enabling factors included wealth index, place of residence (urban or rural), and having health insurance (or private medical aid as it is known in South Africa). The wealth index, which served as a proxy for socioeconomic status, used household-owned assets and housing characteristics aggregated into a Filmer-Pritchett asset wealth index. The 16 variables used to compute the index were housing type, water and sanitation services, and ownership of 13 assets; namely, ownership of a fridge, television, stove, mobile phone, radio, Digital Versatile Disk (DVD) player, washing machine, computer, Digital Satellite Television (DSTV), motorcar, vacuum cleaner, telephone (landline) and internet access.

Need factors comprised hypertension, diabetes, high cholesterol, cardiovascular disease, having accessed health care in the past 5 years, self-reported vision problems and clinician-assessed vision loss. Hypertension was defined as having either their measured systolic blood pressure > =140 mmHg, diastolic blood pressure > =90 mmHg or a self-report of currently taking medication to lower blood pressure [21]. Diabetes and high cholesterol were each based on self-report of having ever been diagnosed with these conditions by a medical professional. A history of cardiovascular disease indicated an admission of previous heart failure, heart disease or myocardial infarction. Having accessed health care in the past 5 years was measured by a self-report of the last time a participant assessed public or private health care such as from a hospital, clinic or health care provider. Self-reported vision problems were based on having difficulty seeing objects, seeing at night or having peripheral vision difficulty. Vision loss was assessed by the clinician who conducted the general physical examination.

### Data analysis

Data were analysed in Stata 15.0. (StataCorp, Texas, USA 2016). The analyses applied sample weights to adjust for unequal probabilities of selection and nonresponse. To investigate the association of all the predisposing, enabling, and need factors on having had an eye examination, the analytic sample comprised 3320 individuals with non-missing responses to the eye examination variables and other independent variables. The distribution of demographic and all other independent variable characteristics did not differ between the analytic sample (mean age: 39.8, 64.9% females, 27.4% ever had an eye examination) and the sample of individuals with missing data (mean age: 39.4, 64.4% females, 26.8% had an eye examination). Characteristics of the sample were presented and differences between males and females were tested using a chi-square analysis. The number of years since the participants’ most recent eye examination was presented by categories of the independent variables. Confidence intervals that did not overlap were used to detect the statistical significance between pairs of point estimates. Andersen’s model was applied in multiple logistic regression models, where a hierarchical approach was used to add the predisposing, enabling, and need factors sequentially. Model 1 included only the predisposing factors, Model 2 the predisposing and enabling factors and Model 3 included the predisposing, enabling, and need factors. The outcome of the multiple logistic regression models was having ever had an eye examination by a medical professional. We detected evidence of effect modification by sex and therefore the multiple logistic regression models were further stratified by sex.

## Results

### Description of the sample

Out of a total of 3320 respondents, two-third were females (64.9%), with a mean age of 39.8 ± 18.1 years (Table 1). The majority of participants were of African descent (70.5%). More than half of the participants lived in urban centers (59.8%). In terms of the availability of health insurance, only 12.9% of the participants had insurance cover. There were significant sex differences in age (*p* = 0.015), ethnicity (*p* = 0.027), high risk alcohol use (*p* < 0.001), current smoking (*p* < 0.001), wealth index (*p* = 0.001), having accessed health care in the past 5 years (*p* = 0.004), and self-reported vision problems (*p* = 0.006). Also, there were significantly higher proportion of 30–44-year olds among the female sample than among the male sample. Further, significantly more males than females were high risk alcohol users (29.8 and 12.7% respectively) and current smokers (32.8 and 12.8%, respectively). In addition, there were significantly more males than females in the highest wealth quintile (14.8 and 10.3%, respectively). Again, having accessed healthcare in the past 5 years (male: 43.7% and females: 48.9%) and self-reported vision problems (male: 17.2% and females: 21.1%) were both significantly more prevalent among females than males.

**Table 1:** Description of the sample

	Total (*N* = 3320)	Male (*N* = 1165, 35.1%)	Female (*N* = 2155, 64.9%)	
	% (frequency)	% (frequency)	% (frequency)	*p*-value for linearity
**Predisposing factors**				
Age (mean, std. dev.)	39.8(18.1)	39.4(18.2)	40(18)	0.367
Age group (years)				
15–29	37(1228)	38.2(445)	36.3(783)	0.015
30–44	23.6(783)	20.8(242)	25.1(541)	
45–59	23.1(767)	24.9(290)	22.1(477)	
60–74	13(430)	13.5(157)	12.7(273)	
> =75	3.4(112)	2.7(31)	3.8(81)	
Ethnicity				
African	70.5(2341)	68.8(802)	71.4(1539)	0.027
White	2.1(69)	2.9(34)	1.6(35)	
Coloured’ (mixed race)	22.6(751)	22.7(264)	22.6(487)	
Indian	4.8(159)	5.6(65)	4.4(94)	
High risk alcohol use	18.7(621)	29.8(347)	12.7(274)	< 0.001
Current smoker	19.8(657)	32.8(382)	12.8(275)	< 0.001
**Enabling factors**				
Wealth index				
Lowest (1)	23.9(793)	25.2(293)	23.2(500)	0.001
2	22.8(757)	21.4(249)	23.6(508)	
3	23.2(771)	20.9(243)	24.5(528)	
4	18.2(604)	17.9(208)	18.4(396)	
Wealthiest (5)	11.9(395)	14.8(172)	10.3(223)	
Residence				
Rural	40.2(1336)	41.2(480)	39.7(856)	0.407
Urban	59.8(1984)	58.8(685)	60.3(1299)	
Has health insurance	12.9(427)	13.1(153)	12.7(274)	0.731
**Need factors**				
Hypertension	35.9(1193)	36.1(421)	35.8(772)	0.857
Diabetes	7.5(249)	7(81)	7.8(168)	0.379
High cholesterol	4.5(149)	4.4(51)	4.5(98)	0.821
Cardiovascular disease	7.4(245)	6.4(75)	7.9(170)	0.127
Accessed health care in past 5 years	47.1(1563)	43.7(509)	48.9(1054)	0.004
Clinician assessed vision loss	11.5(383)	10.6(123)	12.1(260)	0.195
Self-reported vision problems	19.7(655)	17.2(200)	21.1(455)	0.006

### Eye care utilization among study participants

Table 2 shows the proportion of participants who received their last eye examination within the last 2 years, 3–5 years, over 5 years or who had never had an eye examination. Overall, 73.4% (95% CI: 69.7–76.7) of the participants had never had an eye exam, 17.8% were examined in the last 2 years, 5.1% 3–5 years ago and 3.8% longer than 5 years ago. Eye care utilization increased with age group, where 33.6% of ≥75-year olds had an eye exam within the past 2 years compared to 10.2% of 15–29-year olds. The prevalence of having never had an eye exam was higher among participants in rural areas than in urban areas (81.1% versus 66.9%) and varied by ethnicity (from 17.0% in white adults to 78.7% in African adults). Eye care utilization also increased with respect to high risk alcohol use (15.8% of low-risk alcohol users and 26.0% of high-risk alcohol users received their last eye examination within the last 2 years, as compared to 76.2% low-risk alcohol users and 67.1% of high-risk alcohol users who had never had an eye exam). Eye care utilization also increased with increasing wealth; 85.0% of participants in the lowest quintile never had an eye exam as compared to 42.2% of the wealthiest quintile never having an eye exam. With regard to high cholesterol, 75.3% of participants without high cholesterol had never had an eye exam, compared to 31.8% of those with high cholesterol never having had an eye exam. Variations in having never had an eye exam were observed by the predisposing, enabling and need factors.

**Table 2:** Years since last eye examination

	<=2 years		3–5 years		> 5 years		Never had an eye examination		
	%	95% CI	%	95% CI	%	95% CI	%	95% CI	N
Total	17.8	[15.0–20.9]	5.1	[4.2–6.2]	3.8	[2.9–4.8]	73.4	[69.7–76.7]	3320
**Predisposing factors**									
Age (years)									
15–29	10.2	[7.7–13.5]	2.8	[1.8–4.5]	3.0	[2.0–4.6]	83.9	[79.8–87.3]	1227
30–44	15.9	[12.5–20.1]	5.8	[4.1–8.2]	4.2	[2.7–6.4]	74.0	[69.1–78.4]	783
45–59	25.9	[21.0–31.3]	6.9	[4.9–9.6]	3.6	[2.1–6.3]	63.7	[57.5–69.4]	767
60–74	30.7	[22.4–40.5]	8.2	[5.6–11.9]	5.3	[2.9–9.3]	55.8	[46.7–64.6]	430
> =75	33.6	[21.4–48.5]	5.7	[2.6–11.9]	5.1	[2.3–11.3]	55.6	[42.4–68.0]	112
Sex									
Males	19.8	[16.0–24.3]	5.4	[4.0–7.2]	4.1	[2.8–6.0]	70.6	[65.4–75.4]	1165
Females	16.5	[13.6–19.8]	4.9	[3.8–6.3]	3.5	[2.7–4.7]	75.1	[71.6–78.3]	2154
Ethnicity									
African	14.0	[11.4–17.2]	3.9	[3.1–5.0]	3.3	[2.4–4.5]	78.7	[74.9–82.1]	2340
White	66.7	[48.7–80.8]	7.2	[2.9–17.0]	9.1	[4.0–19.2]	17.0	[7.8–33.1]	69
Coloured’ (mixed race)	23.8	[19.2–29.1]	11.8	[9.3–14.9]	5.0	[3.5–7.1]	59.4	[53.6–64.9]	751
Indian	45.6	[34.2–57.4]	19.7	[8.5–39.4]	6.1	[2.3–15.2]	28.7	[21.2–37.5]	159
High-risk alcohol use									
Low risk (1–3 for men and 1–2 for women)	15.8	[13.2–18.8]	4.9	[3.9–6.1]	3.1	[2.4–4.1]	76.2	[72.8–79.2]	2698
High risk (> = 4 for men and > =3 for women)	26.0	[20.7–32.2]	6.0	[4.1–8.7]	6.7	[4.5–9.8]	61.4	[54.1–68.2]	621
Current smoker									
No	17.3	[14.2–20.8]	4.6	[3.7–5.8]	3.5	[2.6–4.6]	74.6	[70.8–78.1]	2662
Yes	20.1	[15.5–25.7]	7.7	[5.4–10.7]	5.2	[3.2–8.3]	67.1	[61.1–72.5]	657
**Enabling factors**									
Wealth index									
Lowest (1)	8.7	[6.6–11.4]	4.1	[2.7–6.3]	2.2	[1.2–3.8]	85.0	[81.5–87.9]	793
2	9.1	[6.8–12.0]	3.3	[1.8–5.9]	3.0	[1.8–5.0]	84.7	[81.0–87.7]	756
3	13.5	[10.3–17.6]	6.4	[4.5–9.1]	5.8	[3.6–9.1]	74.3	[67.8–79.8]	771
4	27.0	[21.8–32.8]	6.0	[4.2–8.6]	3.7	[2.3–5.9]	63.3	[57.0–69.2]	604
Wealthiest (5)	46.0	[35.5–56.8]	6.9	[4.3–10.7]	4.9	[2.6–9.1]	42.2	[32.0–53.2]	395
Residence									
Rural	12.1	[10.2–14.4]	4.2	[3.1–5.8]	2.5	[1.7–3.7]	81.1	[77.9–83.9]	1335
Urban	22.5	[17.8–28.0]	5.8	[4.6–7.4]	4.8	[3.5–6.5]	66.9	[60.9–72.4]	1984
Has health insurance									
No	12.6	[10.9–14.6]	5.1	[4.2–6.3]	3.6	[2.7–4.7]	78.7	[75.8–81.3]	2893
Yes	47.9	[38.8–57.1]	4.9	[3.2–7.4]	4.9	[2.8–8.3]	42.4	[33.7–51.6]	426
**Need factors**									
Hypertension									
No	14.0	[11.4–17.2]	4.4	[3.4–5.7]	3.5	[2.5–4.9]	78.1	[74.5–81.3]	2126
Yes	25.6	[21.1–30.8]	6.5	[4.9–8.6]	4.4	[3.0–6.5]	63.4	[58.0–68.5]	1193
Self-reported diagnosis of Diabetes									
No	16.3	[13.5–19.4]	4.9	[4.0–6.1]	3.7	[2.9–4.9]	75.1	[71.4–78.4]	3070
Yes	39.1	[30.2–48.8]	7.6	[4.7–12.0]	4.2	[1.9–8.8]	49.1	[40.0–58.2]	249
High cholesterol - self-reported									
No	16.6	[14.0–19.6]	4.6	[3.8–5.6]	3.5	[2.7–4.6]	75.3	[71.8–78.4]	3170
Yes	43.3	[30.0–57.6]	15.1	[8.6–25.1]	9.8	[4.8–19.2]	31.8	[21.1–44.8]	149
Cardiovascular disease									
No	17.5	[14.7–20.7]	5.0	[4.1–6.1]	3.4	[2.6–4.5]	74.0	[70.3–77.4]	3074
Yes	20.5	[13.9–29.2]	6.2	[3.6–10.5]	7.8	[4.3–13.7]	65.5	[56.6–73.3]	245
Accessed health care in the past 5 years									
No	12.8	[10.3–15.8]	2.4	[1.6–3.6]	3.0	[2.1–4.3]	81.8	[78.2–84.9]	1756
Yes	23.4	[19.6–27.8]	8.1	[6.6–9.9]	4.7	[3.4–6.4]	63.8	[59.2–68.2]	1563
Clinician assessed vision loss									
No	16.3	[13.3–19.8]	4.9	[4.0–6.0]	3.8	[3.0–5.0]	75.0	[71.0–78.6]	2936
Yes	30.3	[24.4–36.9]	6.8	[4.3–10.7]	3.2	[1.7–5.9]	59.7	[53.6–65.5]	383
Self-reported vision problems									
No	16.2	[13.1–19.8]	4.1	[3.3–5.2]	3.8	[2.9–5.1]	75.9	[71.8–79.5]	2664
Yes	25.0	[20.8–29.8]	9.5	[6.9–13.0]	3.5	[2.2–5.5]	62.0	[56.3–67.4]	655

### Factors associated with eye care utilization

Table 3 presents the results of the multiple logistic regression models, showing the factors associated with ever having an eye examination. Model 1, with predisposing factors, showed that older age (compared with age 15–29 years: age 30–44, OR = 1.85, *p* < 0.001; age 45–59, OR = 3.04, *p* < 0.001; age 60–74, OR = 3.59, *p* < 0.001; age ≥ 75, OR = 4.28, *p* < 0.001), ethnicity (compared with African descent: white descent, OR = 12.76, *p* < 0.001; mixed-race, OR = 2.61, *p* < 0.001; Indian descent, OR = 10.94, *p* < 0.001), and high risk alcohol use (OR = 2.05, *p* < 0.001) were significantly associated with eye care utilization (i.e. having had an eye examination).

**Table 3:** Multiple logistic regression of ever having an eye examination and related factors

	Model 1			Model 2			Model 3		
	OR	95% CI (OR)	*p*-value	OR	95% CI (OR)	*p*-value	OR	95% CI (OR)	*p*-value
**Predisposing factors**									
Age group									
15–29	Ref	–	–	ref	–	–	ref	–	–
30–44	1.85	[1.36–2.51]	< 0.001	1.80	[1.3–2.5]	< 0.001	1.76	[1.27–2.43]	0.001
45–59	3.04	[2.24–4.13]	< 0.001	2.86	[2.09–3.91]	< 0.001	2.13	[1.47–3.07]	< 0.001
60–74	3.59	[2.49–5.18]	< 0.001	4.04	[2.8–5.85]	< 0.001	2.74	[1.81–4.14]	< 0.001
> =75	4.28	[2.61–7.02]	< 0.001	5.01	[3.09–8.12]	< 0.001	3.22	[1.82–5.7]	< 0.001
Sex									
Male	Ref	–	–	ref	–	–	ref	–	–
Female	0.89	[0.7–1.14]	0.370	0.89	[0.69–1.14]	0.349	0.80	[0.61–1.04]	0.090
Ethnicity									
African	Ref	–	–	ref	–	–	ref	–	–
White	12.76	[5.28–30.84]	< 0.001	4.37	[1.73–11.05]	0.002	4.71	[1.8–12.36]	0.002
Coloured’ (mixed race)	2.61	[1.84–3.7]	< 0.001	1.97	[1.38–2.82]	< 0.001	1.87	[1.32–2.65]	< 0.001
Indian	10.94	[5.97–20.03]	< 0.001	8.65	[3.39–22.05]	< 0.001	7.67	[3.17–18.53]	< 0.001
High risk alcohol use	2.05	[1.44–2.92]	< 0.001	1.84	[1.32–2.55]	< 0.001	1.83	[1.3–2.56]	0.001
Current smoking	0.76	[0.53–1.09]	0.134	0.88	[0.63–1.24]	0.478	0.82	[0.59–1.15]	0.253
**Enabling factors**									
Wealth index									
Lowest (1)				ref	–	–	ref	–	–
2				0.96	[0.67–1.4]	0.845	0.93	[0.63–1.38]	0.716
3				1.74	[1.11–2.71]	0.015	1.75	[1.11–2.76]	0.017
4				2.24	[1.49–3.39]	< 0.001	2.23	[1.49–3.36]	< 0.001
Wealthiest (5)				2.50	[1.41–4.45]	0.002	2.49	[1.38–4.48]	0.003
Urban residence				1.17	[0.85–1.62]	0.325	1.10	[0.8–1.51]	0.558
Has health insurance				2.27	[1.49–3.45]	< 0.001	2.19	[1.44–3.33]	< 0.001
**Need factors**									
Hypertension							1.06	[0.8–1.39]	0.696
Diabetes							1.75	[1.15–2.66]	0.009
High cholesterol							2.51	[1.44–4.38]	0.001
Cardiovascular disease							1.27	[0.88–1.83]	0.209
Accessed health care in the past 5 years							2.42	[1.91–3.07]	< 0.001
Clinician assessed vision loss							1.14	[0.78–1.67]	0.506
Self-reported vision problems							1.51	[1.05–2.16]	0.026

Model 1 included predisposing variables as a univariate model; Model 2 included predisposing and enabling variables; Model 3 included predisposing, enabling, and need variables

*OR* Odds ratio, *CI* confidence interval, *n* number of participants, *ref* reference group

In Model 2, which included predisposing and enabling factors, the associations with predisposing factors were similar to that of Model 1. Among the enabling factors, wealth index (compared with lowest quintile 1: wealth quintile 3, OR = 1.74, *p* = 0.015; wealth quintile 4, OR = 2.24, *p* < 0.001; wealthiest quintile 5, OR = 2.50, *p* = 0.002), and having health insurance (OR = 2.27, *p* < 0.001) were significantly associated with increased odds of having had an eye examination.

The final Model 3 incorporated all predisposing, enabling and need factors. After statistical adjustment, age (compared with age 15–29 years: age 30–44, OR = 1.76, *p* = 0.001; age 45–59, OR = 2.13, *p* < 0.001; age 60–74, OR = 2.74, *p* < 0.001; age ≥ 75, OR = 3.22, *p* < 0.001), ethnicity (compared with African descent: white descent, OR = 4.71, *p* = 0.002; mixed race, OR = 1.87, *p* < 0.001; Indian descent, OR = 7.67, *p* < 0.001), high risk alcohol use (OR = 1.83, *p* = 0.001), wealth index (compared with lowest wealth quintile 1: wealth quintile 3, OR = 1.75, *p* = 0.017; wealth quintile 4, OR = 2.23, *p* < 0.001; wealthiest [quintile 5], OR = 2.49, *p* = 0.003), health insurance (OR = 2.19, *p* < 0.001), diabetes (OR = 1.75, *p* = 0.009), high cholesterol (OR = 2.51, *p* = 0.001), having assessed healthcare in the past 5 years (OR = 2.42, *p* < 0.001), and self-reported vision problems (OR = 1.51, *p* = 0.026) were significantly associated with eye care utilization.

### Sex and eyecare utilization

Table 4 shows the multiple logistic regression models of ever having an eye examination and its associated factors by sex. Among males, older age (compared with age 15–29 years: age 30–44, OR = 2.05, *p* = 0.009; age 60–74, OR = 1.94, *p* = 0.030; age ≥ 75, OR = 2.98, *p* = 0.039), ethnicity (compared with African descent: white descent, OR = 8.18, *p* = 0.005; mixed-race, OR = 2.38, *p* < 0.001; Indian descent, OR = 12.00, *p* < 0.001), high risk alcohol use (OR = 2.37, *p* = 0.001), wealth index (compared with lowest wealth quintile 1: wealth quintile 3, OR = 2.62, *p* = 0.002), health insurance (OR = 4.20, *p* < 0.001), diabetes (OR = 3.14, *p* = 0.006), high cholesterol (OR = 2.91, *p* = 0.045) and having assessed healthcare in the past 5 years (OR = 2.91, *p* < 0.001) were significantly associated with eye care utilization.

**Table 4:** Multiple logistic regression of ever having an eye examination and related factors by sex

	Males			Females		
	OR	95% CI (OR)	*p*-value	OR	95% CI (OR)	*p*-value
**Predisposing factors**						
Age group						
15–29	ref	–	–	ref	–	–
30–44	2.05	[1.19–3.51]	0.009	1.59	[1.09–2.31]	0.016
45–59	1.68	[0.97–2.91]	0.066	2.52	[1.64–3.86]	< 0.001
60–74	1.94	[1.07–3.53]	0.030	3.36	[1.94–5.83]	< 0.001
> =75	2.98	[1.06–8.4]	0.039	3.58	[1.78–7.18]	< 0.001
Ethnicity						
African	ref	–	–	ref	–	–
White	8.18	[1.87–35.77]	0.005	3.79	[1.4–10.27]	0.009
Coloured’ (mixed race)	2.38	[1.51–3.75]	< 0.001	1.76	[1.15–2.69]	0.009
Indian	12.00	[4.12–34.94]	< 0.001	6.77	[2.77–16.57]	< 0.001
High risk alcohol use	2.37	[1.45–3.88]	0.001	1.39	[0.84–2.3]	0.196
Current smoking	0.87	[0.56–1.36]	0.544	0.71	[0.45–1.12]	0.139
**Enabling factors**						
Wealth index						
Lowest (1)	ref	–	–	ref	–	–
2	0.97	[0.53–1.79]	0.924	0.89	[0.58–1.38]	0.613
3	2.62	[1.41–4.86]	0.002	1.43	[0.83–2.48]	0.197
4	1.54	[0.83–2.85]	0.168	2.54	[1.56–4.14]	< 0.001
Wealthiest (5)	1.79	[0.78–4.12]	0.170	2.75	[1.41–5.34]	0.003
Urban residence	0.86	[0.55–1.35]	0.504	1.32	[0.9–1.93]	0.156
Has health insurance	4.20	[2.13–8.26]	< 0.001	1.66	[1.07–2.57]	0.024
**Need factors**						
Hypertension	1.04	[0.69–1.58]	0.847	1.02	[0.71–1.47]	0.899
Diabetes	3.14	[1.39–7.08]	0.006	1.48	[0.92–2.39]	0.106
High cholesterol	2.91	[1.03–8.24]	0.045	2.33	[1.34–4.07]	0.003
Cardiovascular disease	1.22	[0.59–2.53]	0.597	1.31	[0.86–2.01]	0.211
Accessed health care in past 5 years	2.91	[1.95–4.36]	< 0.001	2.23	[1.7–2.93]	< 0.001
Clinician assessed vision loss	1.78	[0.91–3.48]	0.092	0.90	[0.55–1.46]	0.658
Self-reported vision problems	1.34	[0.76–2.36]	0.308	1.63	[1.08–2.44]	0.019

*OR* Odds ratio, *CI* confidence interval, *n* number of participants, *ref* reference group

Among females, older age (compared with age 15–29 years: age 30–44, OR = 1.59, *p* = 0.016; age 45–59, OR = 2.52, *p* < 0.001; age 60–74, OR = 3.36, *p* < 0.001; age ≥ 75, OR = 3.58, *p* < 0.001), ethnicity (compared with African descent: white descent, OR = 3.79, *p* = 0.009; mixed race, OR = 1.76, *p* = 0.009; Indian descent, OR = 6.77, *p* < 0.001), wealth index (compared with lowest wealth index 1: wealth quintile 4, OR = 2.54, *p* < 0.001; wealthiest quintile 5, OR = 2.75, *p* = 0.003), health insurance (OR = 1.66, *p* = 0.024), high cholesterol (OR = 2.33, *p* = 0.003), having assessed healthcare in the past 5 years (OR = 2.23, *p* < 0.001), and self-reported vision problems (OR = 1.63, *p* = 0.019) were significantly associated with eye care utilization.

## Discussion

This study investigated the eye care utilization pattern in the South African population and its association with related factors using data from SANHANES-1. We observed that almost 75% of study participants have never had an eye examination while about a tenth had their eyes examined at least once in the last 2 years. We also found age [12], ethnicity [22], alcohol use [23], wealth [24], availability of health insurance [25], diabetes [26] and self-reported vision problems [27] to be significantly associated with eye care utilization. Sex was not associated with eye care utilization. However, further stratification by sex showed that factors associated with eye care utilization differed slightly between males and females.

### Association between eye care utilization and related factors

We found age to be significantly associated with eye care utilization. The older one gets, the more likely they are to seek eye care [28]. This is because as most populations age, there is an anticipated proportionate increase in the risk of developing eye diseases [29]; most of which are caused by chronic diseases such as hypertension and diabetes [5]. Similar findings have been observed across several studies [7, 30–32].

Ethnicity was associated with eye care utilization. Africans were less likely to seek eye care than whites or Indians. Similar findings have been reported by Zhang et al [33] and Orr et al [22]. This observation is likely due to lower socioeconomic status such as education and low-income levels and limited access to insurance policies among some ethnic groups [34, 35]. Numerous studies [22, 36, 37] have found Caucasians to be generally more inclined to seeking eye services as compared to different ethnicities such as Africans or African-Americans. The absence of health insurance among Africans may be due to lack of job-based insurance, low income and general lack of employment.

High-risk alcohol use was found to be associated with eye care utilization. Heavy alcohol drinking leads to temporary blurred vision and may cause long term visual changes. Heavy drinkers also have limited ability to read small prints [23] and are more likely to seek eye care solutions. Interestingly, we observed that smokers were not likely to utilize eye care services, which was also reported by Aljied et al [30]. Smokers generally have a poor attitude towards preventive health [38] even though they are more at risk of developing macular degeneration [39–41], glaucoma and cataract [42] than the general population [43]. Another possible reason could be that since people who smoke may have poor health [44] and other health-related conditions such as respiratory disorders, they may place less priority on utilizing eye care services (as compared to general health services) due to the relatively lower symptomatic effects on vision.

Wealth was found to be significantly associated with eye care utilization in our population. Generally, the more affluent one is, the more likely they are to seek eye care services and possibly, the higher their ability to afford these eye care services. Mashige et al [6] and Ntsoane et al [8] reported similar findings. Several other studies [25, 29, 35] have reported a lack of or inadequate financial resources as a major barrier to seeking eye care. This is because wealthy people are often capable of bearing the cost of better and quality eye care services as compared to people of low socioeconomic status. According to Krisberg [45], wealth is a lifelong determinant of health and directly linked to access to health insurance. We found that people who had access to health insurance were more likely to seek eye care services. This is because benefits from a health insurance package eliminate the burden of paying for medical services upfront and affords one the choice to seek various healthcare services including eye care. This is similar to the findings by Keeffe et al [46], Jiang et al [47] and Chou et al [48].

A significant association was found between having diabetes and eye care utilization. This may be attributable to efforts to comply with recommendations that individuals with diabetes receive an eye exam every 1 to 2 years [49]. Persons with diabetes are at risk of developing diabetic retinopathy after some years of having the disease and so it is generally recommended that they have an eye exam at least once every 2 years. Olusanya et al [12] and Wagner et al. [23] also report similar findings.

Persons with self-reported vision problems were more likely to utilize eye care services. Ahmad et al [50] reported that, if one generally had symptoms of blurredness or any vision-related problems, there is a higher probability for them to seek eye care. Schaumberg et al [51] also reported that the presence of eye problems associated with cataracts was likely to make one seek eye care services.

### Barriers to eye care utilization

Our study shows that nearly three-quarters of South Africans had never had an exam. Olusanya et al [12] and Vela et al [5] also found similar statistics. This may be attributed to several factors (including those discussed above) that could serve as barriers in utilizing eye care services. For instance, we observed that more rural than urban respondents had never had an exam. In South Africa, eye care services are readily available in urban centers and may be generally lacking in rural communities. Underutilization of eye-care services in South Africa may be attributed to the lack of ocular health promotional structures and activities in the primary health care system [52].

### Sex and eyecare utilization

We observed that some factors associated with eye care utilization differed between males and females. For instance, it was observed that females with self-reported vision problems were significantly more likely to get an exam than men with vision problems. The reason for this trend could be that females tend to be more sensitive and so would visit the hospital at the smallest change in vision as compared to men. Another reason for this trend could be the risky lifestyle of denial of weakness and rejecting help as qualities of masculinity [53]. For instance, a man may perceive a change in body function but would not see the need to seek care, hoping it resolves. Boerma et al [54] and Bertakis et al [55] reported similar findings, of females being more likely to report self-assessed vision changes and seek care than men.

Females in the highest wealth quintiles were significantly more likely to seek care than men in the highest wealth quintiles. Wealth is a stronger enabler of care-seeking for women than for men. Wealthy women have the perception of autonomy and are more inclined to participate actively in their own eye (health) care decisions [56] without restriction.

Among participants with health insurance, men were significantly more likely to utilize eye care services than women [57]. This could be attributed to women generally experiencing fewer vision problems as compared to men. Current research shows that estrogen appears to have some protective effect against diabetes [58], cardiovascular [59] and other diseases in women, and therefore are less likely to experience visual problems associated with chronic diseases. Another plausible reason for this trend is that women think about health, and they do more about it in terms of prevention, eating healthier, and are more likely to have health insurance and a regular source of health care as compared to men.

Alcohol use was a strong factor associated with eye care use in men but not in women. Generally, the intensity of heavy drinking is greater in men than women [60], thus they experience more blurred vision problem and are inclined to utilize eye care services.

In general, utilization of eye care services was found to be associated with predisposing, enabling and need factors as explained by Anderson’s model of healthcare utilization. Regarding predisposing factors, utilization of eye care services was associated with age, ethnicity and high-risk alcohol use. Also, enabling factors such as wealth and rural residence were associated with utilizing eye care services. However, the only need factor associated with the utilization of eye care services was self-reported vision problems. Thus, the Andersen Model helped to identify factors driving disparities in eyecare utilization as well as characteristics of persons (aged ≥15 years) who were more likely to utilize eye care services in South Africa.

The strength of our study is that it is a national survey and so allows for trends and patterns of eye care utilization to be examined over time and to be generalized across the entire population. Our study had some limitations. First, the eye care utilization data is self-informed and direct data on eye exam or the presence of disease at the time of examination may be subject to recall bias. A second limitation of this study was the use of data from 2012, which is relatively old. However, this study is the most recent bio-behavioral population-based survey that contains data on eye care utilization as well as clinical examinations. This allowed for the effect of a wide range of predisposing, enabling and need variables to be investigated. Repeat rounds of the survey have delayed due to difficulty in securing funding and revisions of the current research instrumentation. Nonetheless, this data primarily serves as the baseline for assessing subsequent population-based studies. Also, data from our study will assist in the confirmation of changes in eye care utilization patterns in South Africa.

## Conclusion

In conclusion, we found an alarmingly low patronage of eye care services in the South African population. Age, wealth, ethnicity, health insurance, diabetes, high cholesterol, self-reported vision loss, and access to and utilization of general medical care were associated with the level of utilization of eye care services in South Africa. Thus, there is a need for a more effective public health promotion program to address the individual and socio-economic barriers hindering the uptake of eye care services among South Africans. There should be an improved mobile clinic system spanning the rural and far-reaching areas of South Africa to improve eye health coverage (through better and reliable governmental health policies).

## Supplementary information

**Additional file 1.**

**Additional file 2.**

**Additional file 3.**

## Data Availability

The dataset(s) supporting the conclusions of this article is (are) available on request from the HSRC.

## Abbreviations

DSTV: Digital Satellite Television
DVD: Digital Versatile Disk
EAs: Enumeration Areas
HHs: Households
HSRC: Human Sciences Research Council
ICEE: International Centre for Eyecare Education
REC: Research Ethics Committee
SANHANES: South African National Health and Nutrition Examination Survey
