# Supplementary material for: Eye care utilization pattern in South Africa: results from SANHANES-1
Source: BMC Health Serv Res. 2020 Aug 17;20:756. doi: 10.1186/s12913-020-05621-8 (PMC7430111; doi:10.1186/s12913-020-05621-8)
Supplement: Supplementary file 1 — Additional file 1. [file 12913_2020_5621_MOESM1_ESM.pdf]

BARCODE

**Individual Questionnaire Number:** ______________________

# THE SOUTH AFRICAN NATIONAL HEALTH AND NUTRITION EXAMINATION SURVEY, 2011/2012

**(SANHANES-1)**

# Adult Questionnaire: 15 years and older

| 1. Geographic and Interview Particulars | | | | | | | | |
|---|---|---|---|---|---|---|---|---|
| Province | | | | | | | | |
| Enumerator area (EA) | | | | | | | | |
| Visiting point Number (taken from the EA map) | | | | | | | | |
| Visiting point questionnaire number | | | | | | | | |
| **Person number of respondent** | | | | | | | | |

## 2. Interview Details

| | Year | Month | | Day | | Time code | Response code |
|---|---|---|---|---|---|---|---|
| First visit | 2012 | | | | | | |
| Second visit | 2012 | | | | | | |
| Third visit | 2012 | | | | | | |
| Fourth visit | 2012 | | | | | | |
| | | | | | | **Final response code** | |

**Time codes**

1 = Morning till 12h00
2 = 12h01-15h00
3 = 15h01-18h00
4 = 18h01-21h00
5 = 21h01 and later

**Response codes**

1 = Interview completed and clinic appointment made
2 = Interview completed but NO clinic appointment made
3 = Partly completed
4 = Appointment made for interview
5 = Selected respondent not at home
6 = Refusal by respondent / parent / guardian
7 = Other (Specify) ......................

| | | | | | | | | |
|---|---|---|---|---|---|---|---|---|
| **Fieldworker** | **Name** | | | | | | | |
| | **Staff number** | | | | | | | |
| **Interview starting time** | | | | H | H | : | M | M |

| NAME / NICKNAME OF THIS RESPONDENT | ___________________________________ |
|---|---|

**To ensure that we interview the correct person during the follow up survey, would you mind giving us your identity number / passport number?**

| South African Identity number / Passport number | | | | | | | | | | | | | |
|---|---|---|---|---|---|---|---|---|---|---|---|---|---|

| [1] INSTRUCTION TO INTERVIEWER | Record the answers by circling a response or by writing the answer in the space provided. Please note that only one response is allowed per question unless another instruction is given. Please note that coding categories should NOT be read to the participant unless another instruction is given |
|---|---|

| [2] INSTRUCTION TO INTERVIEWER | Tick the appropriate consent forms completed for this respondent |
|---|---|

CONSENT FORM PERSON 18 YEARS AND OLDER…………… ☐

PARENT / GUARDIAN CONSENT FORM 0 TO 17………………… ☐

ASSENT FORM CHILDREN AGED 15 TO 17……………………… ☐

HOUSEHOLD CONSENT TO PARTICIPATE IN THE COHORT…. ☐

## SECTION A BIOGRAPHIC DETAILS OF THE RESPONDENT

| NO. | QUESTIONS AND FILTERS | CODING CATEGORIES | SKIP |
|---|---|---|---|
| 1 | How old were you at your last birthday? | Age in completed years ☐☐ | |
| 2 | Birth date of the respondent | Birth Date D D M M Y Y Y Y | |
| 3 | Is the respondent a male or female? | Male……1<br>Female……2 | |
| 4 | Race of the respondent? | African……1<br>White……2<br>Coloured……3<br>Indian/Asian……4<br>Other (Specify)……5 | |
| 5 | What is your nationality? | South African Citizen……1<br>Non - citizen (permanent resident)……2<br>Non-citizen (refugee)……3<br>Other (Specify)……4 | |
| 6 | How would you describe your present employment situation? | Housewife, homemaker, not looking for work……1<br>Housewife, homemaker, looking for work……2<br>Unemployed, looking for work……3<br>Unemployed, not looking for work……4<br>Work in informal sector, not looking for permanent work……5<br>Sick disabled and unable to work……6<br>Student / pupil/ learner……7<br>Self-employed – Full time (40 hours or more per week)……8<br>Self-employed – Part time (less than 40 hours per week)……9<br>Employed full time (40 hours or more per week)……10<br>Employed part time (less than 40 hours per week)……11<br>Employed (seasonal work)……12<br>Retired……13<br>Other (Specify)……14 | |

| SECTION B | NON COMMUNICABLE DISEASES |
|---|---|

**THE NEXT SET OF QUESTIONS DEAL WITH CHRONIC DISEASES OF LIFESTYLE**

| SECTION B-1 | CARDIOVASCULAR DISEASE | | |
|---|---|---|---|
| NO. | QUESTIONS AND FILTERS | CODING CATEGORIES | SKIP |
| 1 | **I would like to ask you about your family.** Do / did you have a close blood relative (father, mother, brother, sister or child) who has ever had any of the following conditions: | | |
| 1a | High blood Pressure | Yes ....1<br>No ....2<br>Don't know....3 | |
| 1b | Stroke | Yes ....1<br>No ....2<br>Don't know....3 | |
| 1c | Heart attack or angina or chest pain when exerting himself/ herself (walking or exercising)? | Yes ....1<br>No ....2<br>Don't know....3 | <br>-➢2<br>-➢2 |
| 1d | How old was this relative when he/she first had a heart attack, angina or chest pain?<br>(Multiple responses possible - if more than one relative is identified in 1c above) | Younger than 50 years.... 1<br>50 years or older .... 2<br>Don't know.... 3 | |
| 2 | Has a doctor or nurse or health worker at a clinic or hospital told you that you have or have had any of the following conditions: | | |
| 2a | High blood Pressure | Yes ....1<br>No ....2<br>Don't know....3 | |
| 2b | Stroke | Yes ....1<br>No ....2<br>Don't know....3 | |
| 2c | Heart disease | Yes ....1<br>No ....2<br>Don't know....3 | |
| 3a | Do you have or have you had a heart attack or angina (chest pains)? | Yes ....1<br>No ....2<br>Don't know....3 | |
| 3b | Do you have or have you had heart failure? | Yes ....1<br>No ....2<br>Don't know....3 | |
| 3c | Do you have or have you had rheumatic heart disease? | Yes ....1<br>No ....2<br>Don't know....3 | |
| 4a | Have you ever had your blood pressure measured? | Yes ....1<br>No ....2<br>Don't know....3 | <br>-➢5a<br>-➢5a |
| 4b | Have you had your blood pressure measured in the past 12 months? | Yes ....1<br>No ....2<br>Don't know....3 | |
| 4c | Have you ever been told by a doctor/nurse to change your diet or to exercise for high blood pressure? | Yes ....1<br>No ....2<br>Don't know....3 | |
| 4d | Have you ever been given medication by a doctor/nurse for high blood pressure? | Yes ....1<br>No ....2<br>Don't know....3 | <br>-➢5a<br>-➢5a |

## SECTION B-1 CARDIOVASCULAR DISEASE

| NO. | QUESTIONS AND FILTERS | CODING CATEGORIES | SKIP |
|---|---|---|---|
| 4e | Did you take high blood pressure medication in the past month? | Yes....1<br>No....2<br>Don't know....3 | |
| 5a | Have you ever had your <u>blood cholesterol</u> checked? | Yes....1<br>No....2<br>Don't know....3 | |
| 5b | Have you ever been told by a doctor or other health professional that your <u>blood cholesterol</u> level was high? | Yes....1<br>No....2<br>Don't know....3 | |

## SECTION B-2 DIABETES

| NO. | QUESTIONS AND FILTERS | CODING CATEGORIES | SKIP |
|---|---|---|---|
| 1 | Has any <u>close blood relative</u> (father, mother, brother, sister or child) ever been told by a doctor or other health professional that he /she has high blood sugar or sugar diabetes? | Yes....1<br>No....2<br>Don't know....3 | |
| 2 | Have you ever been <u>tested</u> for high blood sugar or sugar diabetes? | Yes....1<br>No....2<br>Don't know....3 | <br>➢4<br>➢4 |
| 3 | How long ago did you have such a test done? | Less than 1 month....1<br>1-12 Months....2<br>1 – 2 Years....3<br>2-5 Years....4<br>More than 5 years....5<br>Refused....6<br>Don't know....7 | |
| 4 | Have you ever been told by a doctor or other health professional that you have high blood sugar or sugar diabetes? | Yes....1<br>No....2<br>Don't know....3 | <br>➢B-3<br>➢B-3 |
| 5 | How old were you when a doctor or other health professional first told you that you had high blood sugar or sugar diabetes? | Age in years.......... ☐☐ | |
| 6 | Which symptoms caused you to go to the doctor when you were diagnosed with high blood sugar or sugar diabetes?<br><br>Multiple responses possible | Frequent urination....1<br>Continuous thirst....2<br>Weight loss....3<br>Tiredness....4<br>Infections....5<br>Coma....6<br>Other (Specify)....7 | |
| 7 | Are you currently taking tablets to lower your blood sugar? | Yes....1<br>No....2<br>Don't know....3 | |
| 8 | Are you currently taking insulin? (Insulin is a medication used to treat diabetes. It is typically taken in the form of injections one or several times per day). | Yes....1<br>No....2<br>Don't know....3 | |
| 9 | Since you were diagnosed with diabetes, have you ever had your eyes examined? This is an examination during which your pupils are usually dilated. It can make you temporarily sensitive to bright light? | Yes....1<br>No....2<br>Don't know....3 | |

| SECTION B-2 | DIABETES | | |
|---|---|---|---|
| NO. | QUESTIONS AND FILTERS | CODING CATEGORIES | SKIP |
| 10 | Has a doctor ever told you that diabetes has affected your eyes, or that you have developed eye diseases due to diabetes? | Yes ....1<br>No ....2<br>Don't know....3 | |
| 11 | Since diagnosis, have you ever been hospitalised for high blood sugar or sugar diabetes? | Yes ....1<br>No ....2<br>Don't know....3 | |
| 12 | How often in the last year (12 months) did you see a doctor/nurse for your high blood sugar or sugar diabetes? | Every month ....1<br>Every 2-3 months....2<br>Every 4-6 months....3<br>Once....4<br>Never....5<br>Refused ....6<br>Don't know....7 | |
| 13 | Beside clinic visits - how often do you check your blood sugar level? | At least once a day ....1<br>At least twice a day ....2<br>At least once a week....3<br>Less frequently....4<br>Never....5<br>Refused ....6<br>Don't know....7 | |
| 14 | Are you a member of any diabetes association or club? | Yes ....1<br>No ....2<br>Don't know....3 | |

| SECTION B-3 | TOBACCO USE | | |
|---|---|---|---|
| NO. | QUESTIONS AND FILTERS | CODING CATEGORIES | SKIP |
| | **The next set of questions are about tobacco use** | | |
| 1 | Have you ever smoked tobacco | Yes, daily....1<br>Yes, less than daily ....2<br>Yes, but not now ....3<br>No, not at all ....4<br>Don't know....5 | <br><br><br>-➢13<br>-➢13 |
| 2 | At what age did you start smoking or using tobacco regularly? | Age (in years)…… ☐☐ | |
| 3 | Do you currently smoke tobacco? | Yes, daily....1<br>Yes, less than daily ....2<br>No, not at all ....3<br>Don't know....4 | <br><br>-➢5<br>-➢5 |
| 4 | For how long have you been smoking tobacco regularly?<br><br>If less than one month – enter "00" for years and "00" for months | Number of years…… ☐☐<br><br>Number of months…… ☐☐ | -➢7 |
| 5 | For how long did you smoke tobacco?<br><br>If less than one month – enter "00" for years and "00" for months | Number of years…… ☐☐<br><br>Number of months…… ☐☐ | |

| SECTION B-3 | TOBACCO USE | | |
|---|---|---|---|
| NO. | QUESTIONS AND FILTERS | CODING CATEGORIES | SKIP |
| 6 | How long is it since you stopped smoking? | Years……………………………………………… ☐☐<br>Months ……………………………………………. ☐☐<br>Weeks……………………………………………… ☐☐<br>Days ………………………………………………. ☐☐ | ->12 |
| 7 | **Current tobacco use** | | |
| 7a | On average how many <u>manufactured cigarettes</u> do you smoke each day / week?<br><br>VERIFY THIS IS THE NUMBER OF CIGARETTES NOT PACKS<br><br>Also let me know if you smoke the product but not every day/week<br>If respondent reports smoking the product but not every (day/week), enter 88. | Per day……………………………………………… ☐☐<br><br>Per week……………………………………………… ☐☐ | |
| 8 | During any visit to a doctor or other health care provider in the past 12 months, were you advised to quit using tobacco? | Yes ........................................1<br>No ........................................2<br>Don't know........................................3 | |
| 9 | During the past 12 months have you tried to stop smoking or using tobacco? | Yes, tried to stop smoking........................................1<br>Yes, tried to stop using snuff/ chewing tobacco........................................2<br>Yes, tried to stop smoking and using snuff/ chewing tobacco........................................3<br>No ........................................4<br>Don't know........................................5 | |
| 10 | In the last 30 days, did you notice any health warnings on tobacco packages? | Yes ........................................1<br>No ........................................2<br>Did not see any tobacco packages........................................3 | |
| 11 | In the last 30 days, have warning labels on tobacco packages led you to think about quitting? | Yes ........................................1<br>No ........................................2<br>Don't know........................................3 | |
| 12 | **Past tobacco use** | | |
| 12a | On average how many <u>manufactured cigarettes</u> did you smoke each day / week?<br>VERIFY THIS IS THE NUMBER OF CIGARETTES NOT PACKS<br><br>Also let me know if you smoke the product but not every day/week<br>If respondent reports smoking the product but not every (day/week), enter 88. | Per day……………………………………………… ☐☐<br><br>Per week……………………………………………… ☐☐ | |
| 13 | How often does anyone smoke inside your home? | Daily........................................1<br>Weekly........................................2<br>Monthly........................................3<br>Less than monthly........................................4<br>Never........................................5<br>Don't know........................................6 | |

## SECTION B-4 OTHER TOBACCO PRODUCTS

| NO. | QUESTIONS AND FILTERS | CODING CATEGORIES | SKIP |
|---|---|---|---|
| | **The next set of questions are about the use of other tobacco products such as hand-rolled cigarettes, pipes full of tobacco, cigars, cheroots, cigarillos, hookah, hubbly bubbly, water pipe, electronic cigarettes, snuff, chewing tobacco, smokeless tobacco.** | | |
| 1 | Have you ever used other tobacco products? | Yes, daily....1<br>Yes, less than daily ....2<br>No, not at all....3<br>Don't know....4 | <br><br>-➢9<br>-➢9 |
| 2 | At what age did you start using other tobacco products regularly? | Age (in years)........ [ ][ ] | |
| 3 | Do you currently use other tobacco products | Yes, daily....1<br>Yes, less than daily ....2<br>No, not at all....3<br>Don't know....4 | <br><br>-➢5<br>-➢5 |
| 4 | For how long have you been using other tobacco products regularly?<br>If less than one month – enter "00" for years and "00" for months | Number of years........ [ ][ ]<br>Number of months........ [ ][ ] | -➢7 |
| 5 | For how long did you use other tobacco products?<br>If less than one month – enter "00" for years and "00" for months | Number of years........ [ ][ ]<br>Number of months........ [ ][ ] | |
| 6 | How long is it since you stopped using other tobacco products? | Years........ [ ][ ]<br>Months ........ [ ][ ]<br>Weeks........ [ ][ ]<br>Days ........ [ ][ ] | -➢8 |
| 7 | **Current tobacco use**<br>On average how many of the following products do you smoke each day / week? Also let me know if you smoke the product but not every day/week<br>If respondent reports smoking the product but not every (day/week), enter 88.<br>If respondent reports not smoking the product, enter 00.<br>**VERIFY THIS IS THE NUMBER OF CIGARETTES NOT PACKS** | | |
| 7a | Hand-rolled cigarettes | Per day........ [ ][ ]<br>Per week........ [ ][ ] | |
| 7b | Pipes full of tobacco | Per day........ [ ][ ]<br>Per week........ [ ][ ] | |
| 7c | Cigars, cheroots, or cigarillos | Per day........ [ ][ ]<br>Per week........ [ ][ ] | |
| 7d | Hookah, hubbly bubbly or water pipe sessions | Per day........ [ ][ ]<br>Per week........ [ ][ ] | |

| SECTION B-4 | OTHER TOBACCO PRODUCTS | |
|---|---|---|
| 7e | Electronic cigarettes | Per day……… [ ][ ]<br>Per week……… [ ][ ] |
| 7f | Any others…<br>Please specify__________________ | Per day……… [ ][ ]<br>Per week……… [ ][ ] |
| 7g | Do you currently use snuff? | Yes, daily……1<br>Yes, less than daily……2<br>No, not at all……3<br>Don't know……4 |
| 7h | Do you currently use chewing tobacco? | Yes, daily……1<br>Yes, less than daily……2<br>No, not at all……3<br>Don't know……4 |
| 7i | Do you currently use other smokeless tobacco? | Yes, daily……1<br>Yes, less than daily……2<br>No, not at all……3<br>Don't know……4 |
| 8 | **Past tobacco use**<br>In the past, did you smoke / use any of the following: | |
| 8a | Hand-rolled cigarettes | Per day……… [ ][ ]<br>Per week……… [ ][ ] |
| 8b | Pipes full of tobacco | Per day……… [ ][ ]<br>Per week……… [ ][ ] |
| 8c | Cigars, cheroots, or cigarillos | Per day……… [ ][ ]<br>Per week……… [ ][ ] |
| 8d | Hookah, hubbly bubbly or water pipe sessions | Per day……… [ ][ ]<br>Per week……… [ ][ ] |
| 8e | Electronic cigarettes | Per day……… [ ][ ]<br>Per week……… [ ][ ] |
| 8f | Any others…<br>Please specify__________________ | Per day……… [ ][ ]<br>Per week……… [ ][ ] |
| 8g | In the past have you used snuff? | Yes, daily……1<br>Yes, less than daily……2<br>No, not at all……3<br>Don't know……4 |
| 8h | In the past have you used chewing tobacco? | Yes, daily……1<br>Yes, less than daily……2<br>No, not at all……3<br>Don't know……4 |

| SECTION B-4 | OTHER TOBACCO PRODUCTS | | |
|---|---|---|---|
| 8i | In the past have you used other smokeless tobacco? | Yes, daily......1<br>Yes, less than daily ......2<br>No, not at all ......3<br>Don't know......4 | |
| 9 | How often does anyone use other tobacco products inside your home? | Daily......1<br>Weekly......2<br>Monthly......3<br>Less than monthly......4<br>Never......5<br>Don't know......6 | |

| SECTION B-5 | PHYSICAL ACTIVITY (GPAQ) | | |
|---|---|---|---|
| NO. | QUESTIONS AND FILTERS | CODING CATEGORIES | SKIP |
| | **The next questions are about the time you spend doing different types of physical activities. You need to think about the time that you spend doing both vigorous and moderate activities in a usual week**<br>• 'vigorous-intensity activities' are activities that require strenuous physical effort and cause large increases in breathing and heart rate<br>• 'moderate-intensity activities' are activities that require moderate effort and cause small increases in breathing and heart rate.<br>You need to think about all the activities you do <u>at home, at work, travelling from place to place and during your spare time.</u> | | |
| 1 | **Work-related Physical Activity (paid or unpaid work outside your own home).**<br>**If you are unemployed, think about the things that keep you physically active during the day.**<br>When answering the following questions, think back over the <u>past 12 months</u> and consider (think of) <u>a usual week.</u> | | |
| 1a | Does your work involve <u>vigorous</u> activities that cause large increases in breathing or heart rate, (like heavy lifting, digging, or heavy construction) for <u>at least 10 minutes</u> at a time? | Yes......1<br>No......2 | -➢2a |
| 1b | In **a** <u>usual week</u>, how many days do you do <u>vigorous</u> activities as part of your work? | Days ...... ☐ | |
| 1c | On **a usual day** on which you do <u>vigorous</u> activities, how much time do you spend doing such work? | Hours ...... ☐☐<br>Minutes ...... ☐☐ | |
| 2a | Does your work involve <u>moderate-intensity</u> activities that cause small increases in breathing and heart rate (like brisk walking or carrying light loads) for <u>at least 10 minutes</u> at a time? | Yes......1<br>No......2 | -➢3 |
| 2b | In <u>a usual week</u>, how many days do you do <u>moderate-intensity</u> activities as part of your work? | Days ...... ☐ | |
| 2c | On **a <u>usual day</u>** on which you do <u>moderate-intensity</u> activities, how much time do you spend doing such work? | Hours ...... ☐☐<br>Minutes ...... ☐☐ | |
| 3 | How long is your usual workday? | Hours ...... ☐☐<br>Minutes ...... ☐☐ | |

| SECTION B-5 | PHYSICAL ACTIVITY (GPAQ) | | |
|---|---|---|---|
| 4 | **Travel-Related Physical Activity:**<br>Other than activities that you've already mentioned, I would like to ask you about the way you travel to and from places (to work, to shopping, to market, to church, etc.). | | |
| 4a | Do you walk or use a bicycle (pedal cycle) for **at least 10 minutes** at a time to get to and from places? | Yes....1<br>No....2 | -➢5 |
| 4b | In **a usual week**, how many days do you walk or cycle for at least 10 minutes to get to and from places? | Days .... ☐ | |
| 4c | On **a usual day,** how much time do you spend walking or cycling for travel? | Hours .... ☐☐<br>Minutes .... ☐☐ | |
| 5 | **Non-Work Related and Leisure Time Physical Activity:**<br>The next questions exclude the work and transport activities you have already mentioned. Now I am going to ask you about activities you do for sport, fitness or recreation in your leisure or spare time**.** | | |
| 5a | Do you do any **<u>vigorous intensity</u>** sport, fitness or recreational activities in your leisure or spare time, that cause large increases in breathing or heart rate (like running or strenuous sports, weightlifting) for **at least 10 minutes** at a time? | Yes....1<br>No....2 | -➢6a |
| 5b | In **a usual week**, how many days do you do **<u>vigorous</u>** activities as part of your leisure or spare time? | Days .... ☐ | |
| 5c | How much time do you spend doing this on **a usual day?** | Hours .... ☐☐<br>Minutes .... ☐☐ | |
| 6a | Do you do any **<u>moderate-intensity</u>** sport, fitness or recreational activities in your leisure or spare time that cause small increases in breathing and heart rate (like brisk walking, cycling or swimming) for **at least 10 minutes** at a time? | Yes....1<br>No....2 | -➢7 |
| 6b | In **a usual week**, how many days do you do **<u>moderate-intensity</u>** activities as part of your leisure or spare time? | Days .... ☐ | |
| 6c | How much time do you spend doing this on **a usual day?** | Hours .... ☐☐<br>Minutes .... ☐☐ | |
| 7 | **Sitting / Resting Activity:**<br>Now I would like to ask you about the time spent sitting or resting, not including sleeping, **in the <u>past 7 days</u>**. This may include time sitting at a desk, riding in a car or taxi, visiting friends, reading, or sitting down to watch television **during working hours and leisure or spare time**. | | |
| 7a | Over the **past 7 days,** how much time did you spend sitting or reclining (lying) on **a usual WEEKDAY** (excluding sleeping)? | Hours .... ☐☐<br>Minutes .... ☐☐ | |
| 7b | Over the **past 7 days,** how much time did you spend sitting or reclining (lying) on **a usual WEEKEND day** (excluding sleeping)? | Hours .... ☐☐<br>Minutes .... ☐☐ | |

| SECTION B-6 | DIET: FOOD FREQUENCY | | |
|---|---|---|---|
| NO. | QUESTIONS AND FILTERS | CODING CATEGORIES | SKIP |
| | **By using your answers to what you have eaten during the past week, we can determine the usual intake of food by South Africans**<br><br>During the past seven days did you eat the following food?<br>If YES, ask how often; IF No, circle 1<br><br>Do not read coding categories to the respondent | | |
| 1 | Processed meat, e.g. sausages, polony, cold cuts, Viennas, Frankfurters, Russians, salami? | None ........ 1<br>Every day ........ 2<br>1-3 times last week ........ 3<br>4-6 times last week ........ 4 | |
| 2 | Food covered with pastry or crumbs, e.g. pies, chicken, beef schnitzel, etc? | None ........ 1<br>Every day ........ 2<br>1-3 times last week ........ 3<br>4-6 times last week ........ 4 | |
| 3 | Food from fast food outlets (take-aways, e.g. pizza, chicken, fish, etc.)? | None ........ 1<br>Every day ........ 2<br>1-3 times last week ........ 3<br>4-6 times last week ........ 4 | |
| 4 | Fried food bought from street vendors, eg. chips, vetkoek, fried chicken, fried fish, etc? | None ........ 1<br>Every day ........ 2<br>1-3 times last week ........ 3<br>4-6 times last week ........ 4 | |
| 5 | Low fat fresh/frozen fish, e.g. hake, without batter or crumbs? | None ........ 1<br>Every day ........ 2<br>1-3 times last week ........ 3<br>4-6 times last week ........ 4 | |
| 6 | Medium fat fresh/frozen, fish, e.g. salmon/mackerel/snoek? | None ........ 1<br>Every day ........ 2<br>1-3 times last week ........ 3<br>4-6 times last week ........ 4 | |
| 7 | Tinned fish, e.g. sardines/pilchards/salmon (excluding tuna)? | None ........ 1<br>Every day ........ 2<br>1-3 times last week ........ 3<br>4-6 times last week ........ 4 | |
| 8 | Food deep fried in oil/fat, e.g. fish, fries/chips, vetkoek, samoosas, doughnuts? | None ........ 1<br>Every day ........ 2<br>1-3 times last week ........ 3<br>4-6 times last week ........ 4 | |
| 9 | Butter, ghee, fat, margarine or oil added to vegetables or other food (like meat) during preparation? | None ........ 1<br>Every day ........ 2<br>1-3 times last week ........ 3<br>4-6 times last week ........ 4 | |
| 10 | Mayonnaise or salad dressing added to food? | None ........ 1<br>Every day ........ 2<br>1-3 times last week ........ 3<br>4-6 times last week ........ 4 | |
| 11 | Cookies, rusks, cakes, pastries? | None ........ 1<br>Every day ........ 2<br>1-3 times last week ........ 3<br>4-6 times last week ........ 4 | |
| 12 | Sweets such as chocolates, fudge or toffees? | None ........ 1<br>Every day ........ 2<br>1-3 times last week ........ 3<br>4-6 times last week ........ 4 | |

| SECTION B-6 | DIET: FOOD FREQUENCY | | |
|---|---|---|---|
| NO. | QUESTIONS AND FILTERS | CODING CATEGORIES | SKIP |
| 13 | Nuts including peanuts? | None ... 1<br>Every day ... 2<br>1-3 times last week ... 3<br>4-6 times last week ... 4 | |
| 14 | Fresh fruit juice, without added sugar? | None ... 1<br>Every day ... 2<br>1-3 times last week ... 3<br>4-6 times last week ... 4 | |
| 15 | Fresh fruit (all the fruit, excluding fruit juices and dried fruit)? | None ... 1<br>Every day ... 2<br>1-3 times last week ... 3<br>4-6 times last week ... 4 | |
| 16 | Dark green leafy or dark yellow vegetables? | None ... 1<br>Every day ... 2<br>1-3 times last week ... 3<br>4-6 times last week ... 4 | |
| 17 | Other vegetables/salad, e.g. cabbage, tomatoes, excluding potatoes? | None ... 1<br>Every day ... 2<br>1-3 times last week ... 3<br>4-6 times last week ... 4 | |
| 18 | Snacks such as chips/crisps, *mazimba*, etc.? | None ... 1<br>Every day ... 2<br>1-3 times last week ... 3<br>4-6 times last week ... 4 | |
| 19 | Salty foods, e.g. salted nuts, biltong, dried sausage, dried salted fish? | None ... 1<br>Every day ... 2<br>1-3 times last week ... 3<br>4-6 times last week ... 4 | |
| 20 | Sweetened cold drink (gas/fizzy cold drink and reconstituted)? | None ... 1<br>Every day ... 2<br>1-3 times last week ... 3<br>4-6 times last week ... 4 | |
| 21 | Sweetened fruit juice? | None ... 1<br>Every day ... 2<br>1-3 times last week ... 3<br>4-6 times last week ... 4 | |
| 22 | Which type of bread spread, i.e. butter/margarine do you usually spread/use on your bread? | None ... 1<br>Margarine Hard type, medium fat (wrapped) ... 2<br>Margarine Hard type, low fat (wrapped) ... 3<br>Margarine Soft type, regular (tub) ... 4<br>Margarine Soft type, medium fat (tub) ... 5<br>Margarine Soft type, light (tub) ... 6<br>Margarine Soft type, extra light (tub) ... 7<br>Cooking fat (eg Holsum) ... 8<br>Animal fat (eg beef, chicken, sausage fat) ... 9<br>Olive oil margarine ... 10<br>Butter (salted) ... 11<br>Butter (unsalted) ... 12<br>Peanut butter ... 13<br>Don't know ... 14 | |
| 23 | Which type of oil do you use in food preparation/as salad dressing most of the time? | None ... 1<br>Canola oil ... 2<br>Olive oil ... 3<br>Soya oil ... 4<br>Sunflower oil ... 5<br>Vegetable oil (mixture) ... 6<br>Other (Specify) ... 7<br>Don't know ... 8 | |

| SECTION B-6 | DIET: FOOD FREQUENCY | | |
|---|---|---|---|
| NO. | QUESTIONS AND FILTERS | CODING CATEGORIES | SKIP |
| 24 | Do you usually eat red meat (beef, mutton and pork) with the fat on, or do you remove the fat from the meat? | Do not eat red meat........1<br>Meat with fat on........2<br>Fat removed from the meat........3 | |
| 25 | Do you usually eat the chicken with the skin, or without the skin? | Do not eat chicken........1<br>With the skin........2<br>Without the skin........3 | |
| 26 | Do you prefer to eat your food usually very salty, lightly salted or not salted? | Very salty........1<br>Lightly salted........2<br>Not salted........3<br>Don't know........4 | |
| 27 | How much butter, fat or margarine do you usually spread on your bread, crackers, or scones? | None........1<br>Very thin / scraped on........2<br>Thin (just covered)........3<br>Medium (nicely covered)........4<br>Thick (see teeth marks)........5<br>Don't know........6 | |
| 28 | How much milk in total do you usually take in per day? | More than 2 cups........1<br>1-2 cups........2<br>½ - 1 cup........3<br>Less than ½ cup........4<br>None........5<br>Don't know........6 | |
| 29 | How many fruits do you usually eat per day? | 4 or more per day........1<br>1-3 per day........2<br>Not every day, but 4 or more per week........3<br>Not every day, but less than 4 per week........4<br>None........5<br>Don't know........6 | |
| 30 | How much fresh/unsweetened fruit juice do you usually drink per day? | More than 2 cups........1<br>1-2 cups........2<br>½ - 1 cup........3<br>Less than ½ cup........4<br>None........5<br>Don't know........6 | |
| 31 | How many portions of vegetables, excluding potatoes, do you usually eat per day? | 4 or more per day........1<br>1-3 per day........2<br>Not every day, but 4 or more per week........3<br>Not every day, but less than 4 per week........4<br>None........5<br>Don't know........6 | |
| 32 | If there are children under five years old in this household, did they eat from the same pot as the rest of the family at the main meal yesterday? | Yes........1<br>No........2<br>No children aged under 5 in household........3 | |

| [3] INSTRUCTION TO INTERVIEWER | Interviewer: Use the example below to help you understand what a standard unit or a standard drink is. One standard drink: |
|---|---|

| | | | |
|---|---|---|---|
| 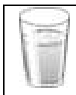 | *A single tot of spirits (whisky, gin, vodka) (e.g. 25ml at 43%)* | 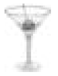 | *A small glass of liqueur or aperitif (e.g. 25ml at 30%)* |

| | | | | | |
|---|---|---|---|---|---|
| 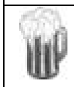 | *1 can of ordinary beer (e.g. 340ml at 5%)* | 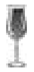 | *1 glass of wine (e.g. 120ml at 12%)* | 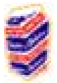 | *Carton of ordinary commercial sorghum beer (e.g. 500ml at 3%)* |

## SECTION B-7 ALCOHOL

| NO. | QUESTIONS AND FILTERS | CODING CATEGORIES | SKIP |
|---|---|---|---|
| 1 | How often did you have a drink containing alcohol in the past 12 months? | Never ........ 0<br>Monthly or less ........ 1<br>2-4 times a month ........ 2<br>2-3 times a week ........ 3<br>4 or more times a week ........ 4 | -➢Filter |
| 2 | How many drinks containing alcohol do you have on a typical day when you are drinking? | 1 or 2........ 0<br>3 or 4........ 1<br>5 or 6........ 2<br>7 , 8 or 9........ 3<br>10 or more ........ 4 | |
| 3 | How often do you have (for men) five or more and (for women) four or more drinks on one occasion? | Never ........ 0<br>Less than monthly ........ 1<br>Monthly ........ 2<br>Weekly ........ 3<br>Daily or almost daily ........ 4 | |

| [4] INSTRUCTION TO INTERVIEWER | MALE / FEMALE FILTER | | |
|---|---|---|---|
| **Female** | ☐ → **Section B-8** | **Male** | ☐ → **Section C** |

## SECTION B-8 PREGNANCY

| NO. | QUESTIONS AND FILTERS | CODING CATEGORIES | SKIP |
|---|---|---|---|
| 1 | Have you ever been pregnant? | Yes ........ 1<br>No ........ 2 | <br>-➢C |
| 2 | During pregnancy, have you ever been told by a doctor or other health professional that you have high blood pressure? | Yes ........ 1<br>No ........ 2<br>Don't know........ 3 | |
| 3 | During pregnancy, have you ever been told by a doctor or other health professional that you have sugar diabetes or sugar disease? | Yes ........ 1<br>No ........ 2<br>Don't know........ 3 | |
| 4 | During pregnancy, did you ever smoke tobacco or use any tobacco products? | Yes ........ 1<br>No ........ 2<br>Don't know........ 3 | <br>-➢7<br>-➢7 |
| 5 | During pregnancy, how many cigarettes did you smoke per day? | Number per day ☐☐ | |

| SECTION B-8 | PREGNANCY | | |
|---|---|---|---|
| NO. | QUESTIONS AND FILTERS | CODING CATEGORIES | SKIP |
| 6 | During which trimester did you smoke tobacco or use tobacco products?<br>Multiple responses possible | During the 1st trimester....1<br>During the 2ndt trimester....2<br>During the 3rd trimester....3 | |
| 7 | During pregnancy, did you ever have a drink containing alcohol? | Yes....1<br>No....2<br>Don't know....3 | <br>➢C<br>➢C |
| 8 | During pregnancy, how many drinks containing alcohol did you have per day? | 1 or 2 per week....1<br>3 or 4 per week....2<br>5 or 6 per week....3<br>1 or 2 per day....4<br>3 or 4 per day....5<br>5 or 6 per day....6<br>7 - 9 per day....7<br>10 or more per day....8 | |
| 9 | During which trimester did you have drink(s) containing alcohol?<br>Multiple responses possible | During the 1st trimester....1<br>During the 2ndt trimester....2<br>During the 3rd trimester....3 | |

## SECTION C — TUBERCULOSIS

**THE FOLLOWING QUESTIONS ARE ABOUT TUBERCULOSIS (TB)**

| SECTION C-1 | TB: KNOWLEDGE AND AWARENESS | | |
|---|---|---|---|
| NO. | QUESTIONS AND FILTERS | CODING CATEGORIES | SKIP |
| 1 | Where did you first learn about tuberculosis or TB?<br>Multiple responses possible | Newspapers and magazines....1<br>Radio....2<br>TV....3<br>Billboards....4<br>Brochures, posters and other printed materials....5<br>Health workers....6<br>Family, friends, neighbours or colleagues....7<br>Religious leaders....8<br>Teachers....9<br>Other (Specify)....10 | |
| 2 | In your opinion, how serious a disease is TB? | Very serious....1<br>Somewhat serious....2<br>Not very serious....3 | |
| 3 | What are the signs and symptoms of TB?<br>Multiple responses possible | Rash....1<br>Cough....2<br>Cough that lasts longer than 3 weeks....3<br>Coughing up blood....4<br>Severe headache....5<br>Nausea....6<br>Weight loss....7<br>Fever....8<br>Fever without clear cause that lasts more than 7 days....9<br>Chest pain....10<br>Shortness of breath....11<br>Ongoing fatigue....12<br>Don't know....13<br>Other (Specify)....14 | |

| SECTION C-1 | TB: KNOWLEDGE AND AWARENESS | | |
|---|---|---|---|
| NO. | QUESTIONS AND FILTERS | CODING CATEGORIES | SKIP |
| 4 | How can a person get TB?<br><br>Multiple responses possible | Through handshakes....1<br>Through the air when a person sneezes or coughs....2<br>Through sharing dishes....3<br>Through eating from the same plate....4<br>Through touching items in public places (doorknobs, handles in transportation, etc)....5<br>Don't know....6<br>Other (Specify)....7 | |
| 5 | How can a person prevent getting TB?<br><br>Multiple responses possible | Avoid shaking hands....1<br>Covering the mouth when coughing or sneezing....2<br>Avoid sharing dishes....3<br>Washing hands after touching items in public places....4<br>Closing windows at home....5<br>Through good nutrition....6<br>By praying....7<br>Don't know....8<br>Other (Specify)....9 | |
| 6 | In your opinion, who could get TB?<br><br>Multiple responses possible | Anybody....1<br>Poor people....2<br>Homeless people....3<br>Alcoholics....4<br>Drug users....5<br>People living with HIV/AIDS....6<br>People who have been in prison....7<br>Don't know....8<br>Other (Specify)....9 | |
| 7 | Can TB be cured? | Yes....1<br>No....2<br>Don't know....3 | <br>-➢9<br>-➢9 |
| 8 | How can someone with TB be cured?<br><br>Multiple responses possible | Herbal remedies....1<br>Home rest without medicine....2<br>Praying....3<br>Specific drugs given by the health centre....4<br>Directly Observed Treatment support (DOTS)....5<br>Don't know....6<br>Other (Specify)....7 | |
| 9 | Are people with TB also HIV positive? | Yes....1<br>No....2<br>Don't know....3 | |
| 10 | Should people with TB also be tested for HIV? | Yes....1<br>No....2<br>Don't know....3 | |

| SECTION C-2 | ATTRIBUTION OF TB | | |
|---|---|---|---|
| NO. | QUESTIONS AND FILTERS | CODING CATEGORIES | SKIP |
| 1 | Have you ever been diagnosed with TB? | Yes....1<br>No....2 | <br>-➢C-3 |
| 2 | Please specify how old you were at the time?<br>If more than once, record the age at the last diagnosis | Age in years.......... ☐☐ | |
| 3 | What do you believe caused you to become sick with TB? | ..........<br>.......... | |
| 4 | Did you go to a traditional healer when you were sick with TB? | Yes....1<br>No....2 | |

| SECTION C-2 | ATTRIBUTION OF TB | | |
|---|---|---|---|
| **NO.** | **QUESTIONS AND FILTERS** | **CODING CATEGORIES** | **SKIP** |
| 5 | Did you get treatment for TB when you had TB? | Yes ....1<br>No ....2 | <br>-➢9 |
| 6 | Did you complete your treatment for TB? | Yes ....1<br>No ....2 | |
| 7 | Did you ever miss your treatment? | Yes ....1<br>No ....2 | <br>-➢9 |
| 8 | Why did you miss your treatment?<br><br>Multiple responses possible<br><br>Write the responses in the space provided below, given to you by the participant and after the interview is completed, circle the coding categories from the list on the right that is the nearest to the responses given by the participant<br><br>__________<br>__________<br>__________<br>__________<br>__________<br>__________<br>__________ | Personal - Feeling<br>I felt better ....1<br>I felt better and gave up ....2<br>I was depressed ....3<br>I was not getting any better / felt worse ....4<br>I was too busy with family obligations....5<br>I didn't like being separated from my home/ family ....6<br>Financial reasons<br>I had to look for a job or money ....7<br>I couldn't afford transport to the clinic....8<br>School / Employment<br>I had to go back to school ....9<br>Employer refused permission to go to the clinic....10<br>I didn't want to lose my job ....11<br>I couldn't get enough time off from work....12<br>Clinic / Health Services<br>The clinic was too far ....13<br>The clinic ran out of medicine / medicine shortage ....14<br>The doctors/ nurses treated me badly ....15<br>Didn't get enough support / no DOTS supporter....16<br>DOTS supporter of the opposite sex ....17<br>Medication<br>I was taking pills for too long.... 18<br>I was taking too many pills....19<br>The pills gave me side effects ....20<br>Other<br>A traditional healer told me to stop ....21<br>A friend / family member told me to stop....22<br>I forgot to come in for TB treatment....23<br>Other (Specify)....24<br>.... | |
| 9 | Does your workplace offer TB treatment support? | Yes ....1<br>No ....2<br>Don't know ....3<br>Don't work ....4 | |

| SECTION C-3 | TB: ATTITUDES AND CARE SEEKING BEHAVIOUR | | |
|---|---|---|---|
| **NO.** | **QUESTIONS AND FILTERS** | **CODING CATEGORIES** | **SKIP** |
| 1 | What would be your reaction if you found out that you have TB?<br><br>Multiple responses possible | Fear ....1<br>Surprise ....2<br>Shame ....3<br>Embarrassment ....4<br>Sadness or hopelessness ....5<br>Other (Specify) ....6 | |
| 2 | Who would you talk to about your illness if you had TB?<br><br>Multiple responses possible | Doctor or other medical worker ....1<br>Spouse....2<br>Parent ....3<br>Children ....4<br>Other family member....5<br>Close friend ....6<br>No one ....7<br>Other (Specify) ....8 | |

| SECTION C-3 | TB: ATTITUDES AND CARE SEEKING BEHAVIOUR | | |
|---|---|---|---|
| NO. | QUESTIONS AND FILTERS | CODING CATEGORIES | SKIP |
| 3 | What would you do if you thought you had symptoms of TB?<br><br>Multiple responses possible | Go to a private hospital ....1<br>Go to a private clinic....2<br>Go to a private doctor....3<br>Go to a public hospital....4<br>Go to a public clinic ....5<br>Go to a traditional healer ....6<br>Other (Specify) ....7 | <br><br><br><br><br>-➢ 8<br>-➢ 8 |
| 4 | If you had symptoms of TB, at what point would you go to a hospital/clinic?<br><br>Multiple responses possible | When treatment on my own does not work....1<br>When symptoms that look like TB last for 3-4 weeks ....2<br>When I realise my symptoms might be related to TB ....3<br>Would not go to a hospital / clinic....4 | <br><br><br>-➢ 8 |
| 5 | What type of hospital/clinic would you go to?<br><br>Read the options and ask the respondent to choose one answer only | Private hospital....1<br>Private clinic ....2<br>Private doctor ....3<br>Public hospital ....4<br>Public clinic....5 | -➢ 6<br>-➢ 6<br>-➢ 6<br>-➢ 7<br>-➢ 7 |
| 6 | Why you would choose a **private** hospital/clinic/doctor and not a public hospital/clinic?<br><br>Multiple responses possible | Quality of service<br>Adequate ....1<br>Inadequate ....2<br>Cost<br>Have health insurance / medical aid ....3<br>Do not have health insurance / medical aid ....4<br>Proximity<br>Near to where I live....5<br>Far from where I live....6<br>Waiting times to be treated<br>Shorter waiting times....7<br>Longer waiting times....8<br>Waiting times for follow up<br>Shorter waiting times....9<br>Longer waiting times....10<br>Physical and medical facilities<br>Adequate ....11<br>Inadequate ....12<br>Other (Specify) ....13 | -➢ 9 |
| 7 | Why you would choose a **public** hospital/clinic and not a private hospital/clinic/doctor?<br><br>Multiple responses possible | Quality of service<br>Adequate ....1<br>Inadequate ....2<br>Cost<br>Have health insurance / medical aid ....3<br>Do not have health insurance / medical aid ....4<br>Proximity<br>Near to where I live....5<br>Far from where I live....6<br>Waiting times to be treated<br>Shorter waiting times....7<br>Longer waiting times....8<br>Waiting times for follow up<br>Shorter waiting times....9<br>Longer waiting times....10<br>Physical and medical facilities<br>Adequate ....11<br>Inadequate ....12<br>Other (Specify) ....13 | -➢ 9 |

## SECTION C-3 TB: ATTITUDES AND CARE SEEKING BEHAVIOUR

| NO. | QUESTIONS AND FILTERS | CODING CATEGORIES | SKIP |
|---|---|---|---|
| 8 | What is the reason that you would not go to a hospital/clinic?<br><br>Multiple responses possible | Not sure where to go ....1<br>Cost ....2<br>Difficulties with transport / distance to clinic....3<br>Do not trust medical workers....4<br>Do not like the attitude of medical workers ....5<br>Cannot leave work (overlapping work hours with medical facility working hours)......6<br>Do not want to find out that something is really wrong ....7<br>Other (Specify) ....8 | |
| 9 | How expensive do you think TB diagnosis and treatment is in this country?<br><br>Note the monetary amount | It is free of charge....1<br>It is reasonably priced ....2<br>It is somewhat / moderately expensive ....3<br>It is very expensive....4<br><br>R________________ | |

## SECTION C-4 TB: ATTITUDES AND STIGMA

| NO. | QUESTIONS AND FILTERS | CODING CATEGORIES | SKIP |
|---|---|---|---|
| 1 | Which statement is closest to your feeling about people with TB disease?<br><br>Read the options and ask the respondent to choose one answer only | I feel sorry for them but I would like to help them .... 1<br>I feel sorry for them but I tend to stay away from these people.... 2<br>It is their problem and I cannot get TB .... 3<br>I fear them because they may infect me .... 4<br>I have no particular feeling .... 5<br>Other (Specify) .... 6 | |
| 2 | In your community, how is a person who has TB usually regarded / treated?<br>Multiple responses possible | Most people reject him or her.... 1<br>Most people are friendly but they generally try to avoid him or her.... 2<br>The community mostly supports him or her .... 3<br>Other (Specify) .... 4 | |
| 3 | Should HIV positive people be concerned about TB? | Yes.... 1<br>No .... 2 | <br>-➢ 5 |
| 4 | Why? | Person with HIV is more likely to develop TB .... 1<br>Don't know.... 2<br>Other (Specify) .... 3 | |
| 5 | Why not? | Person with HIV is not more likely than a person without HIV to develop TB .... 1<br>Don't know.... 2<br>Other (Specify) .... 3 | |

## SECTION C-5 SOURCES OF TB INFORMATION

| NO. | QUESTIONS AND FILTERS | CODING CATEGORIES | |
|---|---|---|---|
| 1 | Are you well informed about TB? | Yes....1<br>No ....2 | |
| 2 | Would you like to have more information about TB? | Yes....1<br>No ....2 | |
| 3 | What are the sources of information that you think can most effectively reach people like you with information on TB?<br><br>Read options and respondent to choose the <u>THREE most</u> effective sources | Newspapers and magazines....1<br>Radio ....2<br>TV ....3<br>Billboards....4<br>Brochures, posters and other printed materials....5<br>Health workers....6<br>Family, friends, neighbours and colleagues ....7<br>Religious leaders....8<br>Teachers....9<br>Other (Specify) ....10 | |

| SECTION D | NUTRITION |
|---|---|

| SECTION D-1 | DIETARY DIVERSITY |
|---|---|

Please describe the foods (meals and snacks and drinks) <u>you ate yesterday</u> during the day and night

| **Breakfast** | **Mid-morning** | **Lunch** | **Mid afternoon** | **Supper** | **After supper** |
|---|---|---|---|---|---|
| | | | | | |

| NO. | QUESTIONS AND FILTERS | CODING CATEGORIES | SKIP |
|---|---|---|---|
| 1 | Did you eat anything (meal or snack) OUTSIDE your home yesterday? | Yes .......... 1<br>No .......... 2 | |
| 2 | Was yesterday a celebration or feast day where you ate special foods or where you ate more, or less than usual? | Yes .......... 1<br>No .......... 2 | |

| 5 INSTRUCTION TO INTERVIEWER | When the interview with the participant is completed, the fieldworker must select the individual food items listed in Section D-1 and link them to the appropriate food groups listed in Section D-2.<br>At the end of the day, the team leader will then double check that the linking has been done correctly |
|---|---|

| **SECTION D-2** | **LIST OF FOOD GROUPS** | | |
|---|---|---|---|
| | **Group** | **Foods** | **Code** |
| 3 | Cereals | Corn/maize/samp, rice, wheat, sorghum, porridge, phutu, bread, pasta, breakfast cereals, oats, Mabella, Morvite, fortified cereals | Yes……..1<br>No..……..2 |
| 4 | White roots and tubers | Potato, white sweet potato | Yes……..1<br>No..……..2 |
| 5 | Yellow/orange vegetables | Carrot, butternut, pumpkin, orange-fleshed sweet potato | Yes……..1<br>No..……..2 |
| 6 | Dark-green leaves | Spinach, imifino, morogo | Yes……..1<br>No..……..2 |
| 7 | Vegetables other than dark-green leafy and yellow/orange | Beetroot, brinjals, broccoli, brussels sprouts, cabbage, cauliflower, gem squash, green beans, onion, peas, tomato, turnip, thepe | Yes……..1<br>No..……..2 |
| 8 | Yellow / orange fruits | Apricot, mango, pawpaw, sweet melon, yellow flesh peach, yellow flesh plums, 100% fruit juice made from these | Yes……..1<br>No..……..2 |
| 9 | Fruit other than yellow / orange fleshed | Apple, avocado, banana, berries, fig, granadilla, grape, grapefruit, guava, lemon, litchi, maroela, melon, orange, naartjie, peach, pear, pineapple, plum, strawberry, watermelon, 100% fruit juice made from these | Yes……..1<br>No..……..2 |
| 10 | Organ meat (offal) | Liver, kidney, heart, spleen, lungs, chicken giblets, malomogudo (offal), intestines | Yes……..1<br>No..……..2 |
| 11 | Meat and poultry (flesh meats) | Beef, goat, lamb, mutton, pork, venison, game, chicken, birds, ostrich, insects, mopani worms, chicken head/feet, sheep head | Yes……..1<br>No..……..2 |
| 12 | Eggs | Any type of egg | Yes……..1<br>No..……..2 |
| 13 | Fish and seafood | Fresh, frozen fish or canned fish (sardines, pilchards, tuna), dried fish, shellfish | Yes……..1<br>No..……..2 |
| 14 | Legumes, nuts and seeds | Dried beans, dried peas, lentils, nuts, peanuts, seeds (or foods made from these e.g. peanut butter) | Yes……..1<br>No..……..2 |
| 15 | Milk and milk products | Milk, sour milk, cheese, yogurt, custard, or any other milk products, or any drinks made with milk eg. cocoa | Yes……..1<br>No..……..2 |
| 16 | Fats and oils | Oils, fats, margarine or butter added to foods or used for cooking | Yes……..1<br>No..……..2 |
| 17 | Sugars and sweets | Sugar, sweets, chocolates, cake and sweetened biscuits, honey, jam, sugar sweetened drinks e.g. cold drinks, sugary foods, sweetened condensed milk | Yes……..1<br>No..……..2 |
| 18 | Spices and condiments | Spices (salt, pepper, etc), condiments (e.g. chutney, tomato sauce) | Yes……..1<br>No..……..2 |
| 19 | Drinks | Coffee, tea | Yes……..1<br>No..……..2 |
| 20 | Drinks | Alcoholic drinks | Yes……..1<br>No..……..2 |
| 21 | Drinks | Cold drinks (except diet cold drinks) and sweetened beverages | Yes……..1<br>No..……..2 |

| SECTION D-3 | DIETARY KNOWLEDGE | | |
|---|---|---|---|
| NO. | QUESTIONS AND FILTERS | CODING CATEGORIES | SKIP |
| 1 | Based on your knowledge, how many servings of fruit and vegetables a day do people need to eat to stay healthy? | Number ………………………………… [ ][ ] | |
| 2 | Based on your knowledge, choose the food that has more sugar? | 100% fruit juice……1<br>Flavoured water……2 | |
| 3 | Based on your knowledge, choose the food that has more fat? | 3a Yoghurt or……1<br>Cream……2 | |
| | | 3b Crisps / chips or……1<br>Popcorn……2 | |
| | | 3c Small bran muffin or……1<br>A slice of whole wheat bread……2 | |
| 4 | Based on your knowledge, choose the food that has more fibre? | 4a Fruit or……1<br>Meat……2 | |
| | | 4b Cornflakes or……1<br>Oats porridge……2 | |
| | | 4c Whole wheat bread or……1<br>White bread……2 | |
| | | 4d Beans or……1<br>Lettuce……2 | |
| | **Do you agree with the following statements?** | | |
| 5 | Starchy food like bread, potatoes and rice make people fat | Yes……1<br>No……2 | |
| 6 | What you eat can make a difference in your chance of becoming fat | Yes……1<br>No……2 | |
| 7 | What you eat can make a difference in your chance of becoming fat and getting diseases like heart disease or cancer…. | Yes……1<br>No……2 | |
| 8 | The things I eat and drink now are healthy, so there is no need for me to make changes | Yes……1<br>No……2 | |
| 9 | How much you eat and drink can make a difference in your chance of becoming fat | Yes……1<br>No……2 | |
| | **Compared to what is healthy do you think your diet is** | | |
| 10 | ……………………………. in **energy** (calories / kilojoules) | Too low……1<br>About right……2<br>Too high……3<br>Other……4 | |
| 11 | ……. in **protein** (meat/ chicken/ fish/ beans/ peas/lentils) | Too low……1<br>About right……2<br>Too high……3<br>Other……4 | |
| 12 | ………………………………. in **fat** (butter/ margarine/ oil) | Too low……1<br>About right……2<br>Too high……3<br>Other……4 | |
| 13 | ……………………………………….. in **sugar and sweets** | Too low……1<br>About right……2<br>Too high……3<br>Other……4 | |

| SECTION D-3 | DIETARY KNOWLEDGE | | |
|---|---|---|---|
| NO. | QUESTIONS AND FILTERS | CODING CATEGORIES | SKIP |
| 14 | ………………………………………………………… in **fruit** | Too low…1<br>About right…2<br>Too high…3<br>Other…4 | |
| 15 | ………………………………………………in **vegetables** | Too low…1<br>About right…2<br>Too high…3<br>Other…4 | |
| 16 | ………….. in **carbohydrates** (bread/ cereals/ rice/ pasta) | Too low…1<br>About right…2<br>Too high…3<br>Other…4 | |

| SECTION D-4 | DIETARY BEHAVIOUR | | |
|---|---|---|---|
| NO. | QUESTIONS AND FILTERS | CODING CATEGORIES | SKIP |
| 1 | How many meals and snacks do you usually have a day? | More than three meals with eating snacks between meals…1<br>Three meals with eating snacks between meals…2<br>Three meals with no eating snacks between meals…3<br>Two meals with eating snacks between meals…4<br>Two meals with no eating snacks between meals…5<br>One meal with eating snacks during the day…6<br>One meal…7<br>Nibble the whole day, no specific meals…8<br>Other (Specify)…9 | |
| 2 | Do you ever eat in places other than at home? | Yes…1<br>No…2 | <br>-➢D-4 |
| 3 | How often do you eat at those places? | More than once a week…1<br>Weekly…2<br>Monthly…3<br>More than once a month…4<br>Other (Specify)…5 | |

| SECTION D-5 | DIETARY PRACTICES | | |
|---|---|---|---|
| NO. | QUESTIONS AND FILTERS | CODING CATEGORIES | SKIP |
| 1 | Please choose one or more factors from the list that influence your choices when you do grocery shopping?<br><br>Multiple responses possible | The price of the food item…1<br>Safety (in terms of hygiene) of the food item…2<br>Taste of the food item…3<br>Convenience…4<br>The nutrient content of the food item…5<br>How well / how long the food item keeps…6<br>How easy the food item is to prepare…7<br>Health considerations…8<br>Other (Specify)…9<br>Don't do grocery shopping…10 | <br><br><br><br><br><br><br><br><br>-➢5 |
| 2 | Do you read food labels when grocery shopping? | Yes…1<br>No…2 | <br>-➢5 |
| 3 | How often do you read food labels? | All the time…1<br>Sometimes…2<br>Other (Specify)…3<br>Never…4 | |
| 4 | Do you understand the information on the food label? | Yes…1<br>No…2 | |

## SECTION D-5 DIETARY PRACTICES

| NO. | QUESTIONS AND FILTERS | CODING CATEGORIES | SKIP |
|---|---|---|---|
| 5 | How often do you wash your hands before handling food? | All the time ........ 1<br>Sometimes........ 2<br>Other (Specify) ........ 3<br>Never ........ 4 | |
| 6 | How often do you wash your hands before eating? | All the time ........ 1<br>Sometimes........ 2<br>Other (Specify) ........ 3<br>Never ........ 4 | |

| 6 INSTRUCTION TO INTERVIEWER | Show the respondent the display card / booklet containing the pictures |
|---|---|

## SECTION D-6 WEIGHT MANAGEMENT

| NO. | QUESTIONS AND FILTERS | CODING CATEGORIES | SKIP |
|---|---|---|---|
| 1 | Choose the picture of the man / woman that you think is…………………………...?<br><br>Enter a response for each option | 1. Very thin………………………………………… ☐<br>2. Normal weight……………………………………… ☐<br>3. Fat……………………………………………… ☐ | |
| 2 | Choose the picture of the man / woman that ………………………………………..?<br><br>Enter a response for each option | 1. You want to look like……………………………….. ☐<br>2. Your husband/ wife / partner want you to look like….. ☐<br>3. Your friends want you to look like……………………… ☐<br>4. Your children want you to look like…………………… ☐ | |
| 3 | Which of the pictures do you think you look the most like ………………………..? | Picture Number ☐ | |
| 4 | How happy are you with your present weight? | Happy ........ 1<br>Somewhat happy........ 2<br>Unhappy ........ 3<br>Other (Specify) ........ 4 | |
| 5 | Do you think you are ……………………? | Underweight ........ 1<br>Normal weight........ 2<br>Overweight ........ 3<br>Other (Specify) ........ 4 | |
| 6 | Would you like to weigh ………………..? | More........ 1<br>Less ........ 2<br>About the right weight for your age ........ 3<br>Stay the same weight as you are now ........ 4<br>Other (Specify) ........ 5 | |
| 7 | Compared with last year do you weigh……… ? | More........ 1<br>Less ........ 2<br>Same weight (kg)........ 3<br>Don't know........ 4 | |
| 8 | Have you ever tried to lose weight? | Yes ........ 1<br>No ........ 2 | |

| SECTION D-6 | WEIGHT MANAGEMENT | | |
|---|---|---|---|
| NO. | QUESTIONS AND FILTERS | CODING CATEGORIES | SKIP |
| 9 | During the past 12 months have you tried to lose weight? | Yes ........ 1<br>No ........ 2 | <br>-➢ 11 |
| 10 | What did you do to lose weight?<br><br>Multiple responses possible | Ate less ........ 1<br>Ate smaller portion sizes ........ 2<br>Increased variety in the diet ........ 3<br>Ate healthier foods........ 4<br>Increased physical activity ........ 5<br>Used weight reducing medication/supplement/product........ 6<br>Other (Specify) ........ 7 | |
| 11 | During the past 12 months have you tried to gain weight? | Yes........ 1<br>No ........ 2 | <br>-➢14 |
| 12 | What did you do to gain weight?<br><br>Multiple responses possible | Ate more food ........ 1<br>Ate bigger portion sizes ........ 2<br>Used supplements ........ 3<br>Limited variety in the diet........ 4<br>Made unhealthy food choices........ 5<br>Reduced physical activity........ 6 | |
| 13 | If you have lost weight over the past 5 years, what factor(s) in your opinion contributed most to the decrease in your weight?<br>Multiple responses possible<br>Do not read the options | Ate less ........ 1<br>Ate smaller portion sizes ........ 2<br>Increased variety in the diet ........ 3<br>Ate healthier foods........ 4<br>Increased physical activity ........ 5<br>Used weight reducing medication/supplement/product........ 6<br>Other (Specify) ........ 7 | |
| 14 | If you have gained weight over the past 5 years, what factor(s) in your opinion contributed most to the increase in your weight?<br>Multiple responses possible<br>Do not read the options | Ate more food ........ 1<br>Ate bigger portion sizes ........ 2<br>Used supplements ........ 3<br>Limited variety in the diet........ 4<br>Made unhealthy food choices........ 5<br>Reduced physical activity........ 6 | |

## SECTION E — PERCEPTIONS OF YOUR GENERAL HEALTH

**Now I am going to ask you questions specifically about your health. The first questions are about your overall health. This is followed by questions on your physical and mental health**

| SECTION E – 1 | HEALTH STATE DESCRIPTIONS | | |
|---|---|---|---|
| NO. | QUESTIONS AND FILTERS | CODING CATEGORIES | SKIP |
| 1 | In general, how would you <u>rate your health today</u> | Very good ........ 1<br>Good ........ 2<br>Moderate........ 3<br>Bad........ 4<br>Very bad ........ 5 | |
| 2 | Overall in the last 30 days, how much difficulty did you have with <u>work or household activities</u>? | None ........ 1<br>Mild ........ 2<br>Moderate........ 3<br>Severe........ 4<br>Extreme / can't do........ 5 | |

| SECTION E – 1 | HEALTH STATE DESCRIPTIONS | | |
|---|---|---|---|
| NO. | QUESTIONS AND FILTERS | CODING CATEGORIES | SKIP |
| | **Now I would like to review the different functions of your body. When answering these questions, I would like you to think about the last 30 days, taking both good and bad days into account. I would like you to consider how much difficulty you have had, on average, while doing the activity in the way that you usually do it. When I ask about difficulty I mean requiring increased effort, discomfort or pain, slowness or changes in the way you do the activity.**<br><br>OVERALL in the last 30 days: | | |
| 3 | How much difficulty did you have with moving around? | None ... 1<br>Mild ... 2<br>Moderate... 3<br>Severe... 4<br>Extreme / can't do... 5 | |
| 4 | How much difficulty did you have in vigorous activities ('vigorous activities' require strenuous physical effort and cause large increases in breathing or heart rate)? | None ... 1<br>Mild ... 2<br>Moderate... 3<br>Severe... 4<br>Extreme / can't do... 5 | |
| 5 | How much difficulty did you have with self-care, such as bathing/washing or dressing yourself? | None ... 1<br>Mild ... 2<br>Moderate... 3<br>Severe... 4<br>Extreme / can't do... 5 | |
| 6 | How much difficulty did you have in taking care of and maintaining your general appearance (for example, grooming, looking neat and tidy)? | None ... 1<br>Mild ... 2<br>Moderate... 3<br>Severe... 4<br>Extreme / can't do... 5 | |
| 7 | How much bodily aches or pains did you have? | None ... 1<br>Mild ... 2<br>Moderate... 3<br>Severe... 4<br>Extreme / can't do... 5 | |
| 8 | How much bodily discomfort did you have? | None ... 1<br>Mild ... 2<br>Moderate... 3<br>Severe... 4<br>Extreme / can't do... 5 | |
| 9 | How much difficulty did you have in your daily life because of your pain? | None ... 1<br>Mild ... 2<br>Moderate... 3<br>Severe... 4<br>Extreme / can't do... 5 | |
| 10 | How much difficulty did you have with concentrating or remembering things? | None ... 1<br>Mild ... 2<br>Moderate... 3<br>Severe... 4<br>Extreme / can't do... 5 | |
| 11 | How much difficulty did you have in learning a new task (eg, learning how to get to a new place, learning a new game, learning a new recipe)? | None ... 1<br>Mild ... 2<br>Moderate... 3<br>Severe... 4<br>Extreme / can't do... 5 | |
| 12 | How much difficulty did you have with personal relationships or participation in the community? | None ... 1<br>Mild ... 2<br>Moderate... 3<br>Severe... 4<br>Extreme / can't do... 5 | |

| SECTION E – 1 | HEALTH STATE DESCRIPTIONS | | |
|---|---|---|---|
| NO. | QUESTIONS AND FILTERS | CODING CATEGORIES | SKIP |
| 13 | How much difficulty did you have in dealing with conflicts and tensions with others? | None ... 1<br>Mild ... 2<br>Moderate ... 3<br>Severe ... 4<br>Extreme / can't do ... 5 | |
| 14 | How much of a problem did you have with sleeping, such as falling asleep, waking up frequently during the night or waking up too early in the morning? | None ... 1<br>Mild ... 2<br>Moderate ... 3<br>Severe ... 4<br>Extreme / can't do ... 5 | |
| 15 | How much of a problem did you have due to not feeling rested and refreshed during the day (for example, feeling tired, not having energy)? | None ... 1<br>Mild ... 2<br>Moderate ... 3<br>Severe ... 4<br>Extreme / can't do ... 5 | |
| 16 | How much of a problem did you have with feeling sad, low or depressed? | None ... 1<br>Mild ... 2<br>Moderate ... 3<br>Severe ... 4<br>Extreme / can't do ... 5 | |
| 17 | How much of a problem did you have with worry or anxiety? | None ... 1<br>Mild ... 2<br>Moderate ... 3<br>Severe ... 4<br>Extreme / can't do ... 5 | |
| | **The next few questions are about your vision and your hearing** | | |
| 18 | When was the last time you had your eyes examined by a medical professional?<br>Enter years or months ago.<br>Enter "00" if less than 1 year | Number of years ago [ ][ ]<br>Don't know... 1<br>Never... 2 | |
| 19 | Do you use eyeglasses or contact lenses to see far away (for example, across the street)? | Yes ... 1<br>No ... 2 | |
| 20 | Do you use eyeglasses or contact lenses to see up close (for example at arms length, like when you are reading)? | Yes ... 1<br>No ... 2 | |
| 21 | In the last 30 days, how much difficulty did you have in seeing and recognising an object or a person you know across the road (from a distance of about 20 meters)?<br>(Respondent should answer, as when wearing glasses/contact lenses if used) | None ... 1<br>Mild ... 2<br>Moderate ... 3<br>Severe ... 4<br>Extreme / can't do ... 5 | |
| 22 | In the last 30 days, how much difficulty did you have in seeing and recognising an object at arm's length (for example, reading)?<br>(Respondent should answer, as when wearing glasses/contact lenses if used) | None ... 1<br>Mild ... 2<br>Moderate ... 3<br>Severe ... 4<br>Extreme / can't do ... 5 | |
| 23 | Do you wear a hearing aid? | Yes ... 1<br>No ... 2 | |
| 24 | In the last 30 days, how much difficulty did you have in: hearing someone talking on the other side of the room in a normal voice<br>(Even with your hearing aid on if you use one)? | None ... 1<br>Mild ... 2<br>Moderate ... 3<br>Severe ... 4<br>Extreme / can't do ... 5 | |

| SECTION E – 1 | HEALTH STATE DESCRIPTIONS | | |
|---|---|---|---|
| NO. | QUESTIONS AND FILTERS | CODING CATEGORIES | SKIP |
| 25 | In the last 30 days, how much difficulty did you have in: hearing what is said in a conversation with one other person in a quiet room<br>(Even with your hearing aid on if you use one)? | None ......1<br>Mild ......2<br>Moderate......3<br>Severe......4<br>Extreme / can't do......5 | |
| | **The next questions ask about difficulties you have due to health conditions. Health conditions include diseases or illnesses, other health problems that may be short or long lasting, injuries, mental or emotional problems, and problems with alcohol or drugs.**<br><br>**Think back over the last 30 days and answer these questions thinking about how much difficulty you had doing the following activities. Some of these questions may seem repetitive, but we do need your attention and it is important to give us answers to each question.**<br><br>**In the last 30 days, how much difficulty did you have in…** | | |
| 26 | Learning a new task (for example, learning how to get to a new place, learning a new game, learning a new recipe)? | None ......1<br>Mild ......2<br>Moderate......3<br>Severe......4<br>Extreme / can't do......5<br>Not applicable......6 | |
| 27 | Making new friendships or maintaining current friendships? | None ......1<br>Mild ......2<br>Moderate......3<br>Severe......4<br>Extreme / can't do......5<br>Not applicable......6 | |
| 28 | Dealing with strangers? | None ......1<br>Mild ......2<br>Moderate......3<br>Severe......4<br>Extreme / can't do......5<br>Not applicable......6 | |
| 29 | Standing for long periods? | None ......1<br>Mild ......2<br>Moderate......3<br>Severe......4<br>Extreme / can't do......5<br>Not applicable......6 | |
| 30 | Taking care of your household responsibilities? | None ......1<br>Mild ......2<br>Moderate......3<br>Severe......4<br>Extreme / can't do......5<br>Not applicable......6 | |
| 31 | Joining in community activities (for example, festivities, religious or other activities) in the same way as anyone else can? | None ......1<br>Mild ......2<br>Moderate......3<br>Severe......4<br>Extreme / can't do......5<br>Not applicable......6 | |
| 32 | Concentrating on doing something for 10 minutes? | None ......1<br>Mild ......2<br>Moderate......3<br>Severe......4<br>Extreme / can't do......5<br>Not applicable......6 | |

| SECTION E – 1 | HEALTH STATE DESCRIPTIONS | | |
|---|---|---|---|
| NO. | QUESTIONS AND FILTERS | CODING CATEGORIES | SKIP |
| 33 | Walking a long distance eg. one kilometre? | None ........ 1<br>Mild ........ 2<br>Moderate ........ 3<br>Severe ........ 4<br>Extreme / can't do ........ 5<br>Not applicable ........ 6 | |
| 34 | Bathing/washing your whole body? | None ........ 1<br>Mild ........ 2<br>Moderate ........ 3<br>Severe ........ 4<br>Extreme / can't do ........ 5<br>Not applicable ........ 6 | |
| 35 | Getting dressed? | None ........ 1<br>Mild ........ 2<br>Moderate ........ 3<br>Severe ........ 4<br>Extreme / can't do ........ 5<br>Not applicable ........ 6 | |
| 36 | Your day to day work? | None ........ 1<br>Mild ........ 2<br>Moderate ........ 3<br>Severe ........ 4<br>Extreme / can't do ........ 5<br>Not applicable ........ 6 | |
| 37 | Moving around inside your home (such as walking across a room)? | None ........ 1<br>Mild ........ 2<br>Moderate ........ 3<br>Severe ........ 4<br>Extreme / can't do ........ 5<br>Not applicable ........ 6 | |
| 38 | Eating (including cutting up your food)? | None ........ 1<br>Mild ........ 2<br>Moderate ........ 3<br>Severe ........ 4<br>Extreme / can't do ........ 5<br>Not applicable ........ 6 | |
| 39 | Getting up from lying down? | None ........ 1<br>Mild ........ 2<br>Moderate ........ 3<br>Severe ........ 4<br>Extreme /can't do ........ 5<br>Not applicable ........ 6 | |
| 40 | Getting to and using the toilet? | None ........ 1<br>Mild ........ 2<br>Moderate ........ 3<br>Severe ........ 4<br>Extreme / can't do ........ 5<br>Not applicable ........ 6 | |
| 41 | In the last 30 days, how much have you been emotionally affected by your health condition(s)? | None ........ 1<br>Mild ........ 2<br>Moderate ........ 3<br>Severe ........ 4<br>Extreme / can't do ........ 5<br>Not applicable ........ 6 | |

## SECTION E – 1 HEALTH STATE DESCRIPTIONS

| NO. | QUESTIONS AND FILTERS | CODING CATEGORIES | SKIP |
|---|---|---|---|
| 42 | Overall, how much did these difficulties interfere with your life? | None ........ 1<br>Mild ........ 2<br>Moderate ........ 3<br>Severe ........ 4<br>Extreme / can't do ........ 5<br>Not applicable ........ 6 | |
| 43 | Besides any vision aids (eyeglasses or contact lenses or hearing aids) mentioned above, do you use any other assistive devices (cane, walker or other) for any difficulties you experience? | Yes ........ 1<br>No ........ 2<br>Don't know ........ 3 | |

## SECTION E-2 PSYCHOLOGICAL DISTRESS

| NO. | QUESTIONS AND FILTERS | CODING CATEGORIES | SKIP |
|---|---|---|---|
| | **The following questions concern how you have been feeling over the past 30 days. select an option for each question that best represents how you have been.** | | |
| 1 | During the last 30 days, about how often did you feel tired out for no good reason? | None of the time ........ 1<br>A little of the time ........ 2<br>Some of the time ........ 3<br>Most of the time ........ 4<br>All of the time ........ 5 | |
| 2 | During the last 30 days, about how often did you feel nervous? | None of the time ........ 1<br>A little of the time ........ 2<br>Some of the time ........ 3<br>Most of the time ........ 4<br>All of the time ........ 5 | |
| 3 | During the last 30 days, about how often did you feel so nervous that nothing could calm you down? | None of the time ........ 1<br>A little of the time ........ 2<br>Some of the time ........ 3<br>Most of the time ........ 4<br>All of the time ........ 5 | |
| 4 | During the last 30 days, about how often did you feel hopeless? | None of the time ........ 1<br>A little of the time ........ 2<br>Some of the time ........ 3<br>Most of the time ........ 4<br>All of the time ........ 5 | |
| 5 | During the last 30 days, about how often did you feel restless or fidgety? | None of the time ........ 1<br>A little of the time ........ 2<br>Some of the time ........ 3<br>Most of the time ........ 4<br>All of the time ........ 5 | |
| 6 | During the last 30 days, about how often did you feel so restless you could not sit still? | None of the time ........ 1<br>A little of the time ........ 2<br>Some of the time ........ 3<br>Most of the time ........ 4<br>All of the time ........ 5 | |
| 7 | During the last 30 days, about how often did you feel depressed? | None of the time ........ 1<br>A little of the time ........ 2<br>Some of the time ........ 3<br>Most of the time ........ 4<br>All of the time ........ 5 | |
| 8 | During the last 30 days, about how often did you feel that everything was an effort? | None of the time ........ 1<br>A little of the time ........ 2<br>Some of the time ........ 3<br>Most of the time ........ 4<br>All of the time ........ 5 | |

## SECTION E-2 — PSYCHOLOGICAL DISTRESS

| NO. | QUESTIONS AND FILTERS | CODING CATEGORIES | SKIP |
|---|---|---|---|
| 9 | During the last 30 days, about how often did you feel so sad that nothing could cheer you up? | None of the time ........ 1<br>A little of the time ........ 2<br>Some of the time ........ 3<br>Most of the time ........ 4<br>All of the time ........ 5 | |
| 10 | During the last 30 days, about how often did you feel worthless? | None of the time ........ 1<br>A little of the time ........ 2<br>Some of the time ........ 3<br>Most of the time ........ 4<br>All of the time ........ 5 | |

## SECTION E-3 — DISTRESS AND TRAUMA FILTER

| NO. | QUESTIONS AND FILTERS | CODING CATEGORIES | SKIP |
|---|---|---|---|
| | Have you ever experienced one of the following events? | | |
| 1 | Military combat | Yes........ 1<br>No........ 2 | |
| 2 | Violent personal assault (sexual assault, physical attack, robbery, mugging) | Yes........ 1<br>No........ 2 | |
| 3 | Being kidnapped | Yes........ 1<br>No........ 2 | |
| 4 | Being taken hostage | Yes........ 1<br>No........ 2 | |
| 5 | Terrorist attack | Yes........ 1<br>No........ 2 | |
| 6 | Incarceration as a prisoner of war or in a concentration camp | Yes........ 1<br>No........ 2 | |
| 7 | Natural or manmade disasters | Yes........ 1<br>No........ 2 | |
| 8 | Severe automobile accidents | Yes........ 1<br>No........ 2 | |
| 9 | Being diagnosed with a life-threatening illness | Yes........ 1<br>No........ 2 | |
| 10 | Inappropriate sexual experiences without threatened or actual violence or injury | Yes........ 1<br>No........ 2 | |
| 11 | Observed the serious injury or unnatural death of another person due to violent assault, accident, war, or disaster or unexpectedly witnessed a dead body or body parts | Yes........ 1<br>No........ 2 | |
| 12 | Learned about a violent personal assault, serious accident, or serious injury experienced by a family member or a close friend? | Yes........ 1<br>No........ 2 | |
| 13 | Learned about the sudden, unexpected death of a family member or a close friend? | Yes........ 1<br>No........ 2 | |
| 14 | Learned that your child has a life-threatening disease? | Yes........ 1<br>No........ 2 | |

| [7] INSTRUCTION TO INTERVIEWER | Distress and Trauma filter |
|---|---|
| **If yes to any of the above** ☐ → **Section E-4** | **If no to all of the above** ☐ → **Section F** |

| SECTION E-4 | DISTRESS AND TRAUMA | | |
|---|---|---|---|
| NO. | QUESTIONS AND FILTERS | CODING CATEGORIES | CODING CATEGORIES |
| | **In the past week, how much trouble have you had with the following symptoms?** | | |
| | | FREQUENCY<br>(If 'not at all' - leave 'severity' column blank) | SEVERITY OF DISTRESS |
| 1 | Have you ever had painful images, memories, or thoughts of the event? | Not at all....0<br>Once only....1<br>2-3 times....2<br>4-5 times....3<br>Every day....4 | None....0<br>Minimal....1<br>Moderate....2<br>Marked....3<br>Extreme....4 |
| 2 | Have you ever had distressing dreams of the event? | Not at all....0<br>Once only....1<br>2-3 times....2<br>4-5 times....3<br>Every day....4 | None....0<br>Minimal....1<br>Moderate....2<br>Marked....3<br>Extreme....4 |
| 3 | Have you felt as though the event were recurring? Was it as if you were reliving it? | Not at all....0<br>Once only....1<br>2-3 times....2<br>4-5 times....3<br>Every day....4 | None....0<br>Minimal....1<br>Moderate....2<br>Marked....3<br>Extreme....4 |
| 4 | Have you been upset by something which reminded you of the event? | Not at all....0<br>Once only....1<br>2-3 times....2<br>4-5 times....3<br>Every day....4 | None....0<br>Minimal....1<br>Moderate....2<br>Marked....3<br>Extreme....4 |
| 5 | Have you been physically upset by reminders of the event? (This includes sweating, trembling, racing heart, shortness of breath, nausea, diarrhea) | Not at all....0<br>Once only....1<br>2-3 times....2<br>4-5 times....3<br>Every day....4 | None....0<br>Minimal....1<br>Moderate....2<br>Marked....3<br>Extreme....4 |
| 6 | Have you been avoiding any thoughts or feelings about the event? | Not at all....0<br>Once only....1<br>2-3 times....2<br>4-5 times....3<br>Every day....4 | None....0<br>Minimal....1<br>Moderate....2<br>Marked....3<br>Extreme....4 |
| 7 | Have you been avoiding doing things or going into situations which remind you of the event? | Not at all....0<br>Once only....1<br>2-3 times....2<br>4-5 times....3<br>Every day....4 | None....0<br>Minimal....1<br>Moderate....2<br>Marked....3<br>Extreme....4 |
| 8 | Have you found yourself unable to recall important parts of the event? | Not at all....0<br>Once only....1<br>2-3 times....2<br>4-5 times....3<br>Every day....4 | None....0<br>Minimal....1<br>Moderate....2<br>Marked....3<br>Extreme....4 |
| 9 | Have you had difficulty enjoying things? | Not at all....0<br>Once only....1<br>2-3 times....2<br>4-5 times....3<br>Every day....4 | None....0<br>Minimal....1<br>Moderate....2<br>Marked....3<br>Extreme....4 |
| 10 | Have you felt distant or cut-off from other people? | Not at all....0<br>Once only....1<br>2-3 times....2<br>4-5 times....3<br>Every day....4 | None....0<br>Minimal....1<br>Moderate....2<br>Marked....3<br>Extreme....4 |

| SECTION E-4 | DISTRESS AND TRAUMA | | |
|---|---|---|---|
| 11 | Have you been unable to have sad or loving feelings? | Not at all....0<br>Once only....1<br>2-3 times....2<br>4-5 times....3<br>Every day....4 | None....0<br>Minimal....1<br>Moderate....2<br>Marked....3<br>Extreme....4 |
| 12 | Have you found it hard to imagine having a long life span fulfilling your goals? | Not at all....0<br>Once only....1<br>2-3 times....2<br>4-5 times....3<br>Every day....4 | None....0<br>Minimal....1<br>Moderate....2<br>Marked....3<br>Extreme....4 |
| 13 | Have you had trouble falling asleep or staying asleep? | Not at all....0<br>Once only....1<br>2-3 times....2<br>4-5 times....3<br>Every day....4 | None....0<br>Minimal....1<br>Moderate....2<br>Marked....3<br>Extreme....4 |
| 14 | Have you been irritable or had outbursts of anger? | Not at all....0<br>Once only....1<br>2-3 times....2<br>4-5 times....3<br>Every day....4 | None....0<br>Minimal....1<br>Moderate....2<br>Marked....3<br>Extreme....4 |
| 15 | Have you had difficulty concentrating? | Not at all....0<br>Once only....1<br>2-3 times....2<br>4-5 times....3<br>Every day....4 | None....0<br>Minimal....1<br>Moderate....2<br>Marked....3<br>Extreme....4 |
| 16 | Have you felt on edge, been easily distracted, or had to stay "on guard"? | Not at all....0<br>Once only....1<br>2-3 times....2<br>4-5 times....3<br>Every day....4 | None....0<br>Minimal....1<br>Moderate....2<br>Marked....3<br>Extreme....4 |
| 17 | Have you been jumpy or easily startled? | Not at all....0<br>Once only....1<br>2-3 times....2<br>4-5 times....3<br>Every day....4 | None....0<br>Minimal....1<br>Moderate....2<br>Marked....3<br>Extreme....4 |

## SECTION F — PERCEPTIONS OF HEALTH CARE SERVICES

***Now I am going to ask you questions about your use of health services***

## SECTION F — HEALTH CARE UTILISATION

| NO. | QUESTIONS AND FILTERS | CODING CATEGORIES | SKIP |
|---|---|---|---|
| 1 | When was the last time that you received health care from a private doctor/hospital/clinic?<br><br>If less than one month ago, enter “00” for years and "00" for months. | Years ago.... [ ][ ]<br>Months ago.... [ ][ ]<br>Don’t know.... 1<br>Never.... 2 | |
| 2 | When was the last time that you received health care from a public hospital/clinic?<br><br>If less than one month ago, enter “00” for years and "00" for months. | Years ago.... [ ][ ]<br>Months ago.... [ ][ ]<br>Don’t know.... 1<br>Never.... 2 | |

| SECTION F | HEALTH CARE UTILISATION | | |
|---|---|---|---|
| NO. | QUESTIONS AND FILTERS | CODING CATEGORIES | SKIP |
| 3 | When was the last time that you needed health care (from a doctor or a hospital)?<br><br>If less than one month ago, enter “00” for years and "00" for months. | Years ago………………………………………… [ ][ ]<br>Months ago……………………………………… [ ][ ]<br>Don't know…………………………………… 1<br>Never………………………………………… 2 | <br><br>-➢ 44<br>-➢ 44 |
| 4 | The last time you needed health care, did you get health care? | Yes ........ 1<br>No ........ 2 | -➢6 |
| 5 | Which reason(s) best explains why you did not get health care?<br><br>Multiple responses possible | Could not afford the cost of the visit........ 1<br>No transport available ........ 2<br>Could not afford the cost of transport ........ 3<br>Previously badly treated........ 4<br>Could not take time off work or had other commitments........ 5<br>The health care provider's drugs or equipment were inadequate ........ 6<br>The health care provider's skills were inadequate........ 7<br>Did not know where to go........ 8<br>Tried but were denied health care ........ 9<br>Thought you were not sick enough........ 10<br>Other (Specify) ........ 11 | |
| 6 | What was the main reason you needed care, even if you did not get care?<br><br>Respondent can select <u>only one</u> main reason for visit | Acute conditions:<br>Diarrhoea........ 1<br>Fever ........ 2<br>Flu........ 3<br>Headaches ........ 4<br>Cough........ 5<br>Stomach ........ 6<br>Muscle ........ 7<br>Non Specific Pain ........ 8<br>Chronic conditions:<br>Chronic pain in your joints/arthritis ( back, neck)........ 9<br>Diabetes or related complications........ 10<br>Heart problems including unexplained pain in your chest………. 11<br>High blood pressure / hypertension........ 12<br>Stroke/sudden paralysis of one side of body ........ 13<br>Cancer........ 14<br>Depression or anxiety ........ 15<br>Communicable diseases: infections, malaria, TB, HIV........ 16<br>Other conditions:<br>Maternal and perinatal conditions (pregnancy)........ 17<br>Nutritional deficiencies ........ 18<br>Surgery........ 19<br>Sleep problems ........ 20<br>Problems with your mouth, teeth or swallowing........ 21<br>Problems with your breathing ........ 22<br>Injury (not work related, see 15 below)........ 23<br>Occupational/work related condition/injury........ 24<br>Other (Specify) ........ 25 | |
| | **<u>IN-PATIENT CARE</u>**<br>**I would like to know if you've had any overnight stays in a hospital or other type of health care facility in the <u>last 12 months</u>.** | | |
| 7 | <u>During the last 12 months</u>, how many different times were you a patient in a hospital for at least one night? | Once........ 1<br>Two – Three times ........ 2<br>Four – Five times........ 3<br>More than Five times........ 4<br>None........ 5<br>Don't know........ 6 | <br><br><br><br>-➢ 25<br>-➢ 25 |

| SECTION F | HEALTH CARE UTILISATION | | |
|---|---|---|---|
| NO. | QUESTIONS AND FILTERS | CODING CATEGORIES | SKIP |
| | **I want to know more about why you needed an overnight stay in a health care facility. Think back to your last overnight hospital stay only.** | | |
| 8 | What type of hospital / facility was it? | Public hospital ....1<br>Private hospital ....2<br>Charity or church run hospital ....3<br>Old persons home ....4<br>Other (Specify) ....5 | |
| 9 | Which reason best describes why you were last hospitalised?<br><br>Respondent can select only one main reason for visit | Acute conditions:<br>Diarrhoea ....1<br>Fever ....2<br>Flu ....3<br>Headaches ....4<br>Cough ....5<br>Stomach ....6<br>Muscle ....7<br>Non Specific Pain ....8<br>Chronic conditions:<br>Chronic pain in your joints/arthritis ( back, neck) ....9<br>Diabetes or related complications ....10<br>Heart problems including unexplained pain in your chest ....11<br>High blood pressure / hypertension ....12<br>Stroke/sudden paralysis of one side of body ....13<br>Cancer ....14<br>Depression or anxiety ....15<br>Communicable diseases: infections, malaria, TB, HIV ....16<br>Other conditions:<br>Maternal and perinatal conditions (pregnancy) ....17<br>Nutritional deficiencies ....18<br>Surgery ....19<br>Sleep problems ....20<br>Problems with your mouth, teeth or swallowing ....21<br>Problems with your breathing ....22<br>Injury (not work related, see 15 below) ....23<br>Occupational/work related condition/injury ....24<br>Other (Specify) ....25<br>………………………… | |
| 10 | How did you get there?<br><br>Multiple responses possible | Private vehicle (own / neighbour / friend) ....1<br>Public transportation ....2<br>Taxi ....3<br>Ambulance or emergency vehicle ....4<br>Bicycle ....5<br>Walked ....6<br>Don't know ....7 | |
| 11 | About how long did it take you to get there? | Hours ……… [ ][ ]<br>Minutes ……… [ ][ ]<br>Don't know ……… 1 | |
| 12 | Who paid for this hospitalization?<br><br>Multiple responses possible | Medical aid ....1<br>Respondent ....2<br>Spouse/partner ....3<br>Son/daughter ....4<br>Other family member ....5<br>Non-family member ....6<br>Hospitalisation was free ....7<br>Other (Specify) ....8 | |

| **SECTION F** | **HEALTH CARE UTILISATION** | | |
|---|---|---|---|
| NO. | QUESTIONS AND FILTERS | CODING CATEGORIES | SKIP |
| | **Thinking about your last [hospital] stay, how much did you or your family/household members have to <u>pay out yourself</u>**<br>Write "00" if the service was free – Write "XX" if a person did not receive medicines or tests | | |
| 13a | Health care providers fees | R [ ][ ][ ] , [ ][ ][ ] | |
| 13b | Medicines | R [ ][ ][ ] , [ ][ ][ ] | |
| 13c | Tests | R [ ][ ][ ] , [ ][ ][ ] | |
| 13d | Transport | R [ ][ ][ ] , [ ][ ][ ] | |
| 13e | Other (Specify) ……………………. | R [ ][ ][ ] , [ ][ ][ ] | |
| 14 | About <u>how much in total</u> did you or your family <u>pay out-of-pocket</u> for this hospitalisation? | R [ ][ ][ ] , [ ][ ][ ]<br>Don't know……………………………………………………………. 1 | |
| 15 | Overall, how <u>satisfied</u> were you with the care you received during your last [hospital] stay? | Very satisfied........1<br>Satisfied........2<br>Neither satisfied nor dissatisfied........3<br>Dissatisfied........4<br>Very dissatisfied........5<br>Don't know........6 | |
| | **I would like to ask you about your impressions of your last overnight stay.**<br>**I would like you to rate your experiences using the following questions.**<br>**How would you rate the following:** | | |
| 16 | The amount of time you <u>waited</u> before being attended to? | Very good........1<br>Good........2<br>Moderate........3<br>Bad........4<br>Very bad........5 | |
| 17 | Your experience of <u>being treated respectfully</u>? | Very good........1<br>Good........2<br>Moderate........3<br>Bad........4<br>Very bad........5 | |
| 18 | How <u>clearly</u> health care providers <u>explained</u> things to you? | Very good........1<br>Good........2<br>Moderate........3<br>Bad........4<br>Very bad........5 | |
| 19 | Your experience of being <u>involved in making decisions</u> for your treatment? | Very good........1<br>Good........2<br>Moderate........3<br>Bad........4<br>Very bad........5 | |
| 20 | The way the health services ensured that you could <u>talk privately</u> to providers? | Very good........1<br>Good........2<br>Moderate........3<br>Bad........4<br>Very bad........5 | |

| SECTION F | HEALTH CARE UTILISATION | | |
|---|---|---|---|
| NO. | QUESTIONS AND FILTERS | CODING CATEGORIES | SKIP |
| 21 | The ease with which you could see a health care provider you were happy with? | Very good....1<br>Good....2<br>Moderate....3<br>Bad....4<br>Very bad....5 | |
| 22 | The cleanliness in the health facility? | Very good....1<br>Good....2<br>Moderate....3<br>Bad....4<br>Very bad....5 | |
| 23 | The availability of medication in the health facility? | Very good....1<br>Good....2<br>Moderate....3<br>Bad....4<br>Very bad....5 | |
| 24 | The availability of tests in the health facility? | Very good....1<br>Good....2<br>Moderate....3<br>Bad....4<br>Very bad....5 | |
| | **OUTPATIENT CARE AND CARE AT HOME**<br>The following questions are about care you received at a hospital, health centre, clinic, private office or at home from a health care worker, but where you did not stay in hospital overnight. | | |
| 25 | Over the past 12 months, did you receive any health care that did not include an overnight stay in hospital? | Yes....1<br>No....2 | <br>-➢ 44 |
| 26 | Over the past 12 months, how many times in total did you receive health care or consultation in an out-patient care situation? | Once....1<br>Two – Three times....2<br>Four – Five times....3<br>More than Five times....4<br>None....5<br>Don't know....6 | <br><br><br><br>-➢ 44<br>-➢ 44 |
| 27 | What was the last (most recent) out-patient health care facility you visited in the past 12 months? | Private doctor's office....1<br>Private clinic or health care facility....2<br>Private hospital....3<br>Public clinic or health care facility....4<br>Public hospital....5<br>Charity or church run clinic....6<br>Charity or church run hospital....7<br>Home visit....8<br>Other (Specify)....9 | |
| 28 | Which was the last (most recent) health care provider you visited? | Medical doctor (including surgeon, gynaecologist, psychiatrist, ophthalmologist)....1<br>Nurse/midwife....2<br>Dentist....3<br>Physiotherapist....4<br>Traditional healer (Inyanga or Isangoma)....5<br>Pharmacist,....6<br>Home health care worker....7<br>Faith healer....8<br>Complementary (homeopath, chiropractor)....9<br>Don't know....10 | |

| NO. | QUESTIONS AND FILTERS | CODING CATEGORIES | SKIP |
|---|---|---|---|
| 29 | Which reason best describes why you needed this visit?<br><br>Respondent to select <u>only one</u> main reason for visit. | Acute conditions:<br>Diarrhoea....1<br>Fever....2<br>Flu....3<br>Headaches....4<br>Cough....5<br>Stomach....6<br>Muscle....7<br>Non Specific Pain....8<br>Chronic conditions:<br>Chronic pain in your joints/arthritis ( back, neck)....9<br>Diabetes or related complications....10<br>Heart problems including unexplained pain in your chest....11<br>High blood pressure / hypertension....12<br>Stroke/sudden paralysis of one side of body....13<br>Cancer....14<br>Depression or anxiety....15<br>Communicable diseases: infections, malaria, TB, HIV....16<br>Other conditions:<br>Maternal and perinatal conditions (pregnancy)....17<br>Nutritional deficiencies....18<br>Surgery....19<br>Sleep problems....20<br>Problems with your mouth, teeth or swallowing....21<br>Problems with your breathing....22<br>Injury (not work related, see 15 below)....23<br>Occupational/work related condition/injury....24<br>Other (Specify)....25 | |
| 30 | How did you get there?<br><br>Multiple responses possible | Private vehicle (own / neighbour / friend)....1<br>Public transportation....2<br>Taxi....3<br>Ambulance or emergency vehicle....4<br>Bicycle....5<br>Walked....6<br>Don't know....7<br>Not applicable....8 | |
| 31 | About how long did it take you to get there? | Hours …… [ ][ ]<br>Minutes…… [ ][ ]<br>Don't know…… 1 | |
| 32 | Who paid for this most recent visit? Anyone else?<br><br>Multiple responses possible | Medical aid....1<br>Respondent....2<br>Spouse/partner....3<br>Son/daughter....4<br>Other family member....5<br>Non-family member....6<br>Voluntary insurance scheme....7<br>It was free....8 | |
| | **Thinking about your <u>last visit</u>, how much did you or your household pay for:**<br>Write "00" if the service was free – Write "XX" if a person did not receive medicines or tests | | |
| 33a | Health care providers fees | R [ ][ ][ ] , [ ][ ][ ] | |
| 33b | Medicines | R [ ][ ][ ] , [ ][ ][ ] | |
| 33c | Tests | R [ ][ ][ ] , [ ][ ][ ] | |
| 33d | Transport | R [ ][ ][ ] , [ ][ ][ ] | |
| 33e | Other (Specify) …………………… | R [ ][ ][ ] , [ ][ ][ ] | |

| SECTION F | HEALTH CARE UTILISATION | | |
|---|---|---|---|
| NO. | QUESTIONS AND FILTERS | CODING CATEGORIES | SKIP |
| 34 | Overall, how satisfied were you with the care you received during your last visit? | Very satisfied ........ 1<br>Satisfied ........ 2<br>Neither satisfied nor dissatisfied ........ 3<br>Dissatisfied ........ 4<br>Very dissatisfied ........ 5 | |
| | I want to know your impressions of your most recent visit for outpatient and home health care.<br>I would like you to rate your experiences using the following questions.<br>For your last visit to a health care provider, how would you rate the following: | | |
| 35 | The amount of time you waited before being attended to? | Very good ........ 1<br>Good ........ 2<br>Moderate ........ 3<br>Bad ........ 4<br>Very bad ........ 5 | |
| 36 | Your experience of being treated respectfully? | Very good ........ 1<br>Good ........ 2<br>Moderate ........ 3<br>Bad ........ 4<br>Very bad ........ 5 | |
| 37 | How clearly health care providers explained things to you? | Very good ........ 1<br>Good ........ 2<br>Moderate ........ 3<br>Bad ........ 4<br>Very bad ........ 5 | |
| 38 | Your experience of being involved in making decisions for your treatment? | Very good ........ 1<br>Good ........ 2<br>Moderate ........ 3<br>Bad ........ 4<br>Very bad ........ 5 | |
| 39 | The way the health services ensured that you could talk privately to providers? | Very good ........ 1<br>Good ........ 2<br>Moderate ........ 3<br>Bad ........ 4<br>Very bad ........ 5 | |
| 40 | The ease with which you could see a health care provider you were happy with? | Very good ........ 1<br>Good ........ 2<br>Moderate ........ 3<br>Bad ........ 4<br>Very bad ........ 5 | |
| 41 | The cleanliness in the health facility? | Very good ........ 1<br>Good ........ 2<br>Moderate ........ 3<br>Bad ........ 4<br>Very bad ........ 5 | |
| 42 | The availability of medication in the health facility? | Very good ........ 1<br>Good ........ 2<br>Moderate ........ 3<br>Bad ........ 4<br>Very bad ........ 5 | |
| 43 | The availability of diagnostics in the health facility? | Very good ........ 1<br>Good ........ 2<br>Moderate ........ 3<br>Bad ........ 4<br>Very bad ........ 5 | |
| | **The last two questions deals with your satisfaction regarding the health system in South Africa. Think about the health care service(s) you received in the last 12 months when answering the questions.** | | |
| 44 | In general, how satisfied were you with how the health care services were run in your area? | Very satisfied ........ 1<br>Satisfied ........ 2<br>Neither satisfied nor dissatisfied ........ 3<br>Dissatisfied ........ 4<br>Very dissatisfied ........ 5 | |
| 45 | How would you rate the way health care was provided in your area? | Very good ........ 1<br>Good ........ 2<br>Moderate ........ 3<br>Bad ........ 4<br>Very bad ........ 5 | |

**Thank you very much for this information. It is invaluable for assessing current health and nutrition trends in South Africa.**

| [8] INSTRUCTION TO INTERVIEWER | Provide the participant with an appointment card to attend the mobile clinic |
|---|---|
| | Record the date and time of the mobile clinic appointment |
| | DATE D D M M Y Y Y Y TIME H H : M M |

| [9] INSTRUCTION TO INTERVIEWER | Record the time at the end of the interview | H H : M M |
|---|---|---|

| [10] INSTRUCTION TO INTERVIEWER | Complete Section G only if you were unable to interview the participant as a result of a refusal |
|---|---|

## SECTION G — REFUSAL PARTICULARS

| NO. | QUESTIONS AND FILTERS | CODING CATEGORIES | SKIP |
|---|---|---|---|
| 1 | At what point did the respondent refuse? | At the gate or door ........ 1<br>After explanation of the survey and the process ........ 2<br>After respondent was identified (before interview)........ 3<br>During the individual interview........ 4<br>After the interview, when requested to take measurements........ 5<br>After the interview, when requested to undergo physical examination ........ 6<br>After the interview, when requested to donate a blood sample........ 7 | |
| 2 | Reason for refusal? | Too busy........ 1<br>Not available........ 2<br>Questionnaire too lengthy ........ 3<br>Not willing to participate in surveys........ 4<br>Security concerns........ 5<br>Privacy / confidentiality........ 6<br>Did not want to take measurements........ 7<br>Did not want undergo physical examination ........ 8<br>Did not want to donate a blood sample........ 9<br>Other (Specify) ........ 10 | |

**OBSERVATIONS:** ……………………………………………………………………………..

…………………………………………………………………………………………………….

……………………………………………………………………………………………………..

……………………………………………………………………………………………………..

| APPOINTED FIELD CHECKER | TEAM LEADER | OFFICE CHECKER |
|---|---|---|
| NAME | NAME | NAME |
| NUMBER | NUMBER | NUMBER |
