# Supplementary material for: Eye care utilization pattern in South Africa: results from SANHANES-1
Source: BMC Health Serv Res. 2020 Aug 17;20:756. doi: 10.1186/s12913-020-05621-8 (PMC7430111; doi:10.1186/s12913-020-05621-8)
Supplement: Supplementary file 2 — Additional file 2. [file 12913_2020_5621_MOESM2_ESM.pdf]

Clinical Examination Form Number: A ………………………….

THE SOUTH AFRICAN NATIONAL HEALTH AND NUTRITION EXAMINATION SURVEY, 2011/2012

(SANHANES-1)

Adult Clinical Examination Form: 15 years and older

	D	D	M	M	Y	Y	Y	Y
**Examination Date**								

	H	H	:	M	M
**Time of arrival**					

	H	H	:	M	M
**Time of departure**					

PASTE

APPOINTMENT

CARD

HERE

Final Response code	
	Full clinical examination completed and bloods collected .... 1<br>Partial clinical examination completed and bloods collected .... 2<br>Only full clinical examination completed .... 3<br>Only partial clinical examination completed .... 4<br>Bloods collected only 5

B. Examination Checklist			Completed		If No, Specify why not
Section A - F	Doctor	History and clinical examination	Y	N	
	Doctor	Fundoscopy – only for diabetic participants	Y	N	
	Doctor	Blood pressure and pulse rate	Y	N	
	Doctor	Cardiovascular Fitness (step test)	Y	N	
Section G	Clinic Assistant	Weight	Y	N	
	Clinic Assistant	Height	Y	N	
	Clinic Assistant	Mid upper arm circumference	Y	N	
	Clinic Assistant	Waist circumference	Y	N	
	Clinic Assistant	Hip circumference	Y	N	
	Clinic Assistant	Triceps Skinfold	Y	N	
	Clinic Assistant	Sub scapular Skinfold	Y	N	
	Clinic Assistant	Biceps Skinfold	Y	N	
	Clinic Assistant	Supra-iliac Skinfold	Y	N	
Section H - J	Nurse	Spirometry	Y	N	
	Nurse	Bioelectrical Impedance	Y	N	
	Nurse	Blood collection	Y	N	

**Clinic Administrator**	**Name**							
	**Staff Number**							

**Doctor**	**Name**							
	**Staff Number**							

**Clinic Assistant**	**Name**							
	**Staff Number**							

**Nurse**	**Name**							
	**Staff Number**							

INSTRUCTIONS	Sections A – F to be completed by the Doctor

Doctor	Start time	H	H	:	M	M

SECTION A — GENERAL PATIENT HISTORY (all participants)

I would like to ask you about your general health (circle as appropriate)

1	When was the last time you had something to eat?	Last night (before midnight) ..... 1<br>06:00 – 08:00 ..... 2<br>08:00 – 10:00 ..... 3<br>10:00 – 12:00 ..... 4<br>12:00 – 14:00 ..... 5<br>14:00 – 16:00 ..... 6<br>16:00 – 18:00 ..... 7<br>18:00 – 20:00 8
2	When was the last time you had something to drink?	Last night (before midnight) ..... 1<br>06:00 – 08:00 ..... 2<br>08:00 – 10:00 ..... 3<br>10:00 – 12:00 ..... 4<br>12:00 – 14:00 ..... 5<br>14:00 – 16:00 ..... 6<br>16:00 – 18:00 ..... 7<br>18:00 – 20:00 8
3	In your opinion, how would you describe your health today?<br>**If completely healthy, go to Question 7**	Completely healthy ..... 1<br>Feeling unwell ..... 2<br>Don't know 3
4	How long have you not been feeling well or had this illness?	One day ..... 1<br>One week ..... 2<br>One month ..... 3<br>Longer 4
5	Do you have a fever today?	Yes ..... 1<br>No 2
6	Have you sought medical care for your illness or health problem?<br>If yes, where have you gone for medical care?<br><br>**Multiple responses possible**	Yes: GP / Private practitioner ..... 1<br>Yes: Clinic ..... 2<br>Yes: Day hospital ..... 3<br>Yes: Other public hospital ..... 4<br>Yes: Private hospital ..... 5<br>No 6
7	Do you have asthma?	Yes ..... 1<br>No 2
8	Do you have any past history of illness?<br>If yes, what history of illness do you have?<br><br>**Multiple responses possible**	Yes: ENT ..... 1<br>Yes: Heart ..... 2<br>Yes: Blood Sugar ..... 3<br>Yes: Chest ..... 4<br>Yes: Abdomen ..... 5<br>Yes: Cancer ..... 6<br>Yes: Other (specify) ..... 7<br>No 8

SECTION A	GENERAL PATIENT HISTORY (all participants)	
9	Is there a family history of illness?<br>If yes, what illness runs in the family?<br><br>**Multiple responses possible**	Yes: Heart .... 1<br>Yes: Blood Sugar .... 2<br>Yes: Chest .... 3<br>Yes: Abdomen .... 4<br>Yes: Cancer .... 5<br>Yes: Other (specify) .... 6<br>No 7
10	Have you had any past surgery?<br>If yes, what surgery have you had?<br><br>**Multiple responses possible**	Yes: ENT .... 1<br>Yes: Heart .... 2<br>Yes: Chest .... 3<br>Yes: Abdomen .... 4<br>Yes: Cancer .... 5<br>Yes: Other (specify) .... 6<br>No 7
11	Have you had any injuries?<br>If yes, what type of injuries did you have?<br><br>**Multiple responses possible**	Yes: Head .... 1<br>Yes: Limbs .... 2<br>Yes: Chest .... 3<br>Yes: Abdomen .... 4<br>No 5
12	Are you currently on any medication?<br>If yes, what type of medication are you currently on?<br><br>**Multiple responses possible**	Yes: Antibiotics .... 1<br>Yes: Anti-inflammatory .... 2<br>Yes: Pain .... 3<br>Yes: Chest .... 4<br>Yes: ENT .... 5<br>Yes: Blood sugar .... 6<br>Yes: Blood pressure .... 7<br>Yes: Diuretics .... 8<br>Yes: Heart disease .... 9<br>Yes: Aspirin (daily) .... 10<br>Yes: Antilipids .... 11<br>Yes: Abdominal illness (diarrhoea or other) .... 12<br>Yes: Depression .... 13<br>Yes: Other mental illness .... 14<br>Yes: Other (specify) .... 15<br>No 16
13	Do you have any physical disabilities?<br><br>**Multiple responses possible**	Yes: Impairment in mobility .... 1<br>Yes: Spinal cord disability .... 2<br>Yes: Brain disability .... 3<br>Yes: Vision disability .... 4<br>Yes: Hearing disability .... 5<br>Yes: Cognitive disability .... 6<br>Yes: Other (specify) .... 7<br>No 8
14	Do you have any mental disabilities?<br><br>**Multiple responses possible**	Yes: Mental disorder .... 1<br>Yes: Neurological disorder .... 2<br>Yes: Learning disability .... 3<br>Yes: Mental retardation .... 4<br>Yes: Other (specify) .... 5<br>No 6

SECTION B	DETAILED PATIENT HISTORY: Over the past 3 months<br>(all participants)

1 GENERAL SYSTEMS (Over the past three months)

1.1	Have you lost weight without dieting?	Yes .... 1<br>No 2
1.2	Have you gained weight?	Yes .... 1<br>No 2
1.3	Has your appetite changed?	Yes: It has increased .... 1<br>Yes: It has decreased .... 2<br>No 3
1.4	Has your eating pattern changed?	Yes: He/she eats more frequently .... 1<br>Yes: He/she eats less often .... 2<br>No 3
1.5	Has your fluid intake changed?	Yes: It has increased .... 1<br>Yes: It has decreased .... 2<br>No 3
1.6	Have you been feeling thirsty more than usual?	Yes .... 1<br>No 2
1.7	Did you have a fever?	Yes .... 1<br>No 2
1.8	Have you felt tired / lethargic?	Yes .... 1<br>No 2
1.9	Did you have night sweats?	Yes .... 1<br>No 2

2 HEAD, EYES, EARS, NOSE, MOUTH AND THROAT (Over the past three months)

2.1	Have you had any headaches?	Yes: Migraine .... 1<br>Yes: Tension .... 2<br>Yes: Cluster .... 3<br>Yes: Other (specify) .... 4<br>No 5
2.2	Have you had repeated episodes of dizziness?	Yes .... 1<br>No 2
2.3	Have you had a seizure?<br><br>**Multiple responses possible**	Yes: Grand mal .... 1<br>Yes: Petit mal .... 2<br>Yes: Other (specify) .... 3<br>No 4
2.4	Have you had any head trauma?	Yes .... 1<br>No 2
2.5	Have you had any of the following eye problems?<br><br>**Multiple responses possible**	Yes: Difficulties in seeing things .... 1<br>Yes: Cannot see at night .... 2<br>Yes: Cannot see from the sides of your eyes .... 3<br>Yes: Discharge (yellow) .... 4<br>Yes: Discharge (watery) .... 5<br>Yes: Discharge (other) .... 6<br>Yes: Other (specify) .... 7<br>No 8

2.6	Have you had any of the following ear problems?<br><br>**Multiple responses possible**	Yes: Ear pain .... 1<br>Yes: Ringing in the ears........ 2<br>Yes: Feels like the room is spinning........ 3<br>Yes: Discharge (yellow)........ 4<br>Yes: Discharge (watery)........ 5<br>Yes: Discharge (bloody)........ 6<br>Yes: Discharge (other)........ 7<br>Yes: Other (specify)........ 8<br>No 9
2.7	Have you had any of the following nose problems?<br><br>**Multiple responses possible**	Yes: Nose bleeds........ 1<br>Yes: Discharge........ 2<br>Yes: Sinus........ 3<br>Yes: Other (specify)........ 4<br>No........ 5
2.8	Have you had any of the following mouth problems?<br><br>**Multiple responses possible**	Yes: Sore throat........ 1<br>Yes: Toothache........ 2<br>Yes: Pain in the mouth or gums........ 3<br>Yes: Other (specify)........ 4<br>No........ 5

3 RESPIRATORY SYSTEM (Over the past three months)

3.1	Have you had any difficulty in breathing / shortness of breath? If yes, what kind of difficulty did you have in breathing?<br><br>**Multiple responses possible**	Yes: Worse at night........ 1<br>Yes: Wheezing........ 2<br>Yes: Stridor........ 3<br>Yes: Other (specify)........ 4<br>No........ 5
3.2	Did you have a cough?	Yes........ 1<br>No........ 2
3.3	If yes, please specify the type of cough?	Dry........ 1<br>Wet........ 2<br>Other (specify)........ 3<br>........
3.4	If yes, please specify the colour of the sputum?	White........ 1<br>Yellow........ 2<br>Green........ 3<br>Blood........ 4<br>Other (specify)........ 5<br>........
3.5	Were you in contact with a person that coughs a lot?	Yes........ 1<br>No........ 2
3.6	Did you have an asthma attack?	Yes........ 1<br>No........ 2

4 CARDIOVASCULAR SYSTEM (Over the past three months)

4.1	Did you have any chest pain?	Yes........ 1<br>No........ 2
4.2	Did you have shortness of breath when lying down?	Yes........ 1<br>No........ 2

SECTION B	DETAILED PATIENT HISTORY: Over the past 3 months (all participants)	
4.3	Did you have swelling of any of the following?	Yes: Hands .... 1<br>Yes: Feet .... 2<br>Yes: Both hands and feet .... 3<br>Yes: Other (specify) .... 4<br>No 5

5 GASTRO-INTESTINAL SYSTEM (Over the past three months)

5.1	Did you have any of the following tummy problems?<br><br>**Multiple responses possible**	Yes: Pain in the stomach .... 1<br>Yes: Nausea .... 2<br>Yes: Vomiting .... 3<br>Yes: Diarrhoea .... 4<br>Yes: Constipation .... 5<br>Yes: Bloody stools .... 6<br>Yes: Yellowing (discolouration) of the eyes .... 7<br>Yes: Problem eating or swallowing .... 8<br>Yes: Other (specify) .... 9<br>No 10

6 GENITO-URINARY SYSTEM (Over the past three months)

6.1	Did you pass urine more than usual?	Yes .... 1<br>No 2
6.2	Did you have pain when passing urine?	Yes .... 1<br>No 2
6.3	Did you have blood in the urine?	Yes .... 1<br>No 2
6.4	Did the colour of your urine differ from what it usually is?	Yes .... 1<br>No 2
6.5	Did you experience any of the following?<br><br>**Multiple responses possible**<br><br>**FOR MEN ONLY**	Yes: Reduced force when passing urine .... 1<br>Yes: Hesitancy to pass urine .... 2<br>Yes: Dribble when passing urine .... 3<br>Yes: Other (specify) .... 4<br>No 5

7 SEXUAL AND REPRODUCTIVE SYSTEM (Over the past three months)

7.1	Did you have any of the following problems?<br><br>**Multiple responses possible**<br><br>**FOR MEN ONLY**	Yes: Urethral discharge “drops” .... 1<br>Yes: Groin swelling .... 2<br>Yes: Testicular swelling .... 3<br>Yes: Other (specify) .... 4<br>No 5
7.2	Did you have any of the following problems?<br><br>**Multiple responses possible**<br>**FOR WOMEN ONLY**	Yes: Vaginal discharge .... 1<br>Yes: Vaginal bleeding (other than menstruation) .... 2<br>Yes: Other (specify) .... 3<br>No 4
7.3	When was the last time you had a pap smear?<br><br>**FOR WOMEN ONLY**	0 – 6 months ago .... 1<br>6 – 12 months ago .... 2<br>1 – 2 years .... 3<br>2 – 4 years ago .... 4<br>5 –10 years ago .... 5<br>More than 10 years ago .... 6<br>Never 7

SECTION B	DETAILED PATIENT HISTORY: Over the past 3 months (all participants)

8 SKIN AND LYMPH NODES (Over the past three months)

8.1	Did you have any of the following skin problems?<br><br>**Multiple responses possible**	Yes: Skin rashes (local)........1<br>Yes: Skin rashes (generalised) ....2<br>Yes: Skin rashes (itchy) ....3<br>Yes: Bruises........4<br>Yes: Skin discolouration........5<br>Yes: Other (specify) ....6<br>No........7
8.2	Did you have any swollen glands?<br>If yes, which of your glands were swollen?<br><br>**Multiple responses possible**	Yes: Neck ....1<br>Yes: Axilla........2<br>Yes: Epitrochlear........3<br>Yes: Other (specify) ....4<br>No........5

9 MUSCULOSKELETAL DISORDERS (Over the past three months)

9.1	Did you have any of the following muscle / bone problems?<br><br>**Multiple responses possible**	Yes: Pain in the joints ....1<br>Yes: Swelling in the joints ....2<br>Yes: Arthritis ....3<br>Yes: Muscle pain........4<br>Yes: Other (specify) ....5<br>No........6

SECTION C	SYSTEMATIC CLINICAL EXAMINATION (all participants)

1 GENERAL SYSTEMS

1.1	Pallor	Yes........1<br>No........2
1.2	Rash<br><br>**Multiple responses possible**	Yes: Scratch marks........1<br>Yes: Erythematous........2<br>Yes: Blanching on pressure........3<br>Yes: Purpuric........4<br>Yes: Macules........5<br>Yes: Papules........6<br>Yes: Vesicles ....7<br>Yes: Impetigo........8<br>No........9
1.3	Jaundice	Yes........1<br>No........2
1.4	Central cyanosis	Yes........1<br>No........2
1.5	Peripheral cyanosis	Yes........1<br>No........2
1.6	Finger clubbing	Yes........1<br>No........2
1.7	General oedema	Yes........1<br>No........2
1.8	Peripheral oedema	Yes........1<br>No........2

SECTION C	SYSTEMATIC CLINICAL EXAMINATION (all participants)	
1.9	Lymphadenopathy<br>If yes, please specify the position<br><br>**Multiple responses possible**	Yes: Neck .... 1<br>Yes: Axilla .... 2<br>Yes: Epitrochlear .... 3<br>No 4
1.10	Hydration<br>If dehydrated, specify the grade / symptoms<br><br>**Multiple responses possible**	Normal .... 1<br>**Dehydrated (mild-moderate)**<br>Restlessness / irritability .... 2<br>Sunken eyes .... 3<br>Headache .... 4<br>Decreased urine output .... 5<br>Skin turgor <2 seconds .... 6<br>**Dehydrated (severe)**<br>Sleepy/lethargic .... 7<br>Sunken eyes .... 8<br>Dizziness or light-headedness .... 9<br>Skin turgor >2 seconds 10
1.11	Dysmorphism	Yes .... 1<br>No 2
1.12	Hearing	Normal .... 1<br>Loss of hearing 2
1.13	If loss of hearing, record which of the following are present<br><br>**Multiple responses possible**	Inability to hear people clearly and fully .... 1<br>Frequent requests for repetition or clarification .... 2<br>Tendency to bluff .... 3<br>Tendency to lip read .... 4<br>None 5
1.14	Vision	Normal .... 1<br>Loss of vision 2
1.15	If loss of vision, record which of the following are present<br><br>**Multiple responses possible**	Blurred vision .... 1<br>Need for more light .... 2<br>Gradual loss of peripheral, or side, vision .... 3<br>Difficulty driving at night .... 4<br>Double vision .... 5<br>Difficulty in reading .... 6<br>Difficulty in distinguishing colours .... 7<br>Straight lines look wavy .... 8<br>Sensitivity to glare 9
1.16	Fundoscopy: Macula<br><br>**Only for diabetics**<br>**Multiple responses possible**	Normal .... 1<br>Abnormal: Oedema .... 2<br>Abnormal: Lipid deposition .... 3<br>Abnormal: Capillary obliteration .... 4<br>Abnormal: Other (specify) .... 5<br>..........
1.17	Fundoscopy: Retina<br><br>**Only for diabetics**<br>**Multiple responses possible**	Normal .... 1<br>Abnormal: Microaneurisms .... 2<br>Abnormal: Intra retinal haemorrhages .... 3<br>Abnormal: Exudates .... 4<br>Abnormal: Neovascularisation .... 5<br>Abnormal: Vitreous haemorrhage .... 6<br>Abnormal: Other (specify) .... 7<br>..........

SECTION C	SYSTEMATIC CLINICAL EXAMINATION (all participants)

2 EARS, NOSE AND THROAT

2.1	Mouth sores	Yes .... 1<br>No 2
2.2	Nasal Discharge<br>If yes, specify the type of discharge<br><br>**Multiple responses possible**	Yes: Yellow .... 1<br>Yes: Watery .... 2<br>Yes: Bloody .... 3<br>Yes: Other .... 4<br>No 5
2.3	Swollen tonsils	Yes .... 1<br>No 2
2.4	Ears<br><br>**Multiple responses possible**	Normal .... 1<br>Infection .... 2<br>No 3

3 RESPIRATORY SYSTEM

3.1	Nasal flare	Yes .... 1<br>No 2
3.2	Chest shape<br><br>**Multiple responses possible**	Normal .... 1<br>Pectus carinatum .... 2<br>Pectus excavatum .... 3<br>Scoliosis .... 4<br>Kyphosis .... 5<br>Gynecomastia (male) .... 6<br>Barrel chest deformity 7
3.3	Rib retraction	Yes .... 1<br>No 2
3.4	Tachypnoea	Yes: >10 years: >20/min .... 1<br>No 2
3.5	Cyanosis	Yes: Central .... 1<br>Yes: Peripheral .... 2<br>No 3
3.6	Trachea displacement	Yes .... 1<br>No 2
3.7	Percussion	Normal .... 1<br>Abnormal: Consolidation .... 2<br>Abnormal: Pleural effusion 3
3.8	Auscultation<br><br>**Multiple responses possible**	Normal .... 1<br>Abnormal: Unequal bilateral air entry .... 2<br>Abnormal: Crepitations .... 3<br>Abnormal: Rhonchi .... 4<br>Abnormal: Wheezing .... 5<br>Abnormal: Consolidation (Bronchial Breathing) 6

4 CARDIOVASCULAR SYSTEM

4.1	Apex<br><br>**Multiple responses possible**	Normal .... 1<br>Visible pulsations .... 2<br>Displaced 3
4.2	Pulse<br><br>**Record Pulse in Section E**	Normal .... 1<br>*> 10 Years: 60-100/min*<br>Tachycardia 2

4.3	Auscultation: Heart sounds<br><br>**Multiple responses possible**	Normal......1<br>Abnormal: Systolic Grade I......2<br>Abnormal: Systolic Grade II......3<br>Abnormal: Systolic Grade III......4<br>Abnormal: Systolic Grade IV......5<br>Abnormal: Diastolic Grade I......6<br>Abnormal: Diastolic Grade II......7<br>Abnormal: Diastolic Grade III......8<br>Abnormal: Diastolic Grade IV......9
4.4	Auscultation: Arrhythmia	Yes: Tachyarrhythmia......1<br>Yes: Bradyarrhythmia......2<br>Yes: Atrial Fibrillation......3<br>No......4

5 GASTRO-INTESTINAL SYSTEM

5.1	Distension	Yes......1<br>No......2
5.2	Tenderness<br>If yes, specify location<br><br>**Multiple responses possible**	Yes: Upper central......1<br>Yes: Lower central......2<br>Yes: Left hypochondrium......3<br>Yes: Right hypochondrium......4<br>Yes: Left inguinal......5<br>Yes: Right inguinal......6<br>Yes: Left flank......7<br>Yes: Right flank......8<br>Yes: Suprapubic......9<br>No......10
5.3	Abdominal mass<br>If yes, specify location<br><br>**Multiple responses possible**	Yes: Upper central......1<br>Yes: Lower central......2<br>Yes: Left hypochondrium......3<br>Yes: Right hypochondrium......4<br>Yes: Left inguinal......5<br>Yes: Right inguinal......6<br>Yes: Left flank......7<br>Yes: Right flank......8<br>Yes: Suprapubic......9<br>No......10
5.4	Organomegaly	Yes: Hepatomegaly......1<br>Yes: Splenomegaly......2<br>No......3
5.5	Renal mass	Yes......1<br>No......2
5.6	Hernias	Yes......1<br>No......2
5.7	Bowel sounds	Normal......1<br>Absent......2<br>Accentuated......3

SECTION C	SYSTEMATIC CLINICAL EXAMINATION (all participants)

6 CENTRAL NERVOUS SYSTEM

6.1	Face, Head shape, neck<br>If abnormal, specify which of the following are present<br><br>**Multiple responses possible**	Normal....1<br>Abnormal: Facial tics....2<br>Abnormal: Bell's palsy....3<br>Abnormal: Padget's disease....4<br>Abnormal: Acromegaly....5<br>Abnormal: Cervical arthritis....6<br>Abnormal: Thyroid....7
6.2	Motor function: gait<br>If abnormal, specify which of the following are present<br><br>**Multiple responses possible**	Normal....1<br>Abnormal: Ataxia....2<br>Abnormal: Spasticity....3<br>Abnormal: Staggering....4
6.3	Motor function: movements<br>If abnormal, specify which of the following are present<br><br>**Multiple responses possible**	Normal....1<br>Abnormal: Unable to sit/stand....2<br>Abnormal: Tremor....3<br>Abnormal: Tics....4<br>Abnormal: Rolling eye movements....5
6.4	Motor function: tone<br>If abnormal, specify which of the following are present<br><br>**Multiple responses possible**	Normal....1<br>Abnormal: Hypotonia....2<br>Abnormal: Hypertonia....3<br>Abnormal: Limited movements....4<br>Abnormal: Uncontrolled movements....5<br>Abnormal: Brudzinski's sign....6
6.5	Motor function: coordination<br>If abnormal, specify which of the following are present<br><br>**Multiple responses possible**	Normal....1<br>Abnormal: Change in walking....2<br>Abnormal: Dropping things more than usual....3<br>Abnormal: Spilling fluids more than usual....4
6.6	Level of consciousness<br>If abnormal, specify which of the following are present<br><br>**Multiple responses possible**	Normal....1<br>Abnormal: Hyperexcitability....2<br>Abnormal: Unresponsiveness....3<br>Abnormal: Drowsiness....4<br>Abnormal: Semi-conscious....5<br>Abnormal: Unconscious....6

7 MUSCULO-SKELETAL SYSTEM

7.1	Muscle weakness	Yes....1<br>No....2
7.2	Bone swellings<br><br>**Multiple responses possible**	Yes: Long bones and joints....1<br>Yes: Ribs....2<br>Yes: Spine....3<br>Yes: Skull....4<br>No....5

SECTION D	**Referral** (identified participants)

PROVISIONAL DIAGNOSIS

**Clinical impression/ provisional diagnosis**					
**Referral to (please tick)**	GP / Private Practitioner	Clinic	Day hospital	Other public hospital	Private hospital
**Referral letter given**			Yes .... 1<br>No 2		

SECTION E	**Blood pressure and pulse rate** (participants 8yrs and older)

	Measurement type	Recorded measurements			Final Blood Pressure
1	Systolic (mmHg)	1 ☐☐☐	2 ☐☐☐	3 ☐☐☐	4 ☐☐☐
2	Diastolic (mmHg)	1 ☐☐☐	2 ☐☐☐	3 ☐☐☐	4 ☐☐☐
3	**PULSE RATE** (bpm)	1 ☐☐☐	2 ☐☐☐	3 ☐☐☐	4 ☐☐☐

SECTION F	**Step fitness test** (participants aged 18-40 years)

Note: *Participants can be excluded from this test on clinical grounds.*

1	Was the step test performed?	Yes .... 1<br>No 2
2	If the test was not done, specify the reason	Cardiac .... 1<br>Respiratory .... 2<br>Other .... 3<br>........
3	AGE	☐☐
4	MAX PULSE RATE FOR AGE **(from the table)**	☐☐☐
5	**PULSE RATE** (bpm)	☐☐☐
6	Systolic (mmHg) **(POST EXERCISE)**	☐☐☐
7	Diastolic (mmHg) **(POST EXERCISE)**	☐☐☐

**Doctor**	**End time**	H H : M M

INSTRUCTIONS	Section G to be completed by the Clinic Assistant

Clinic Assistant	Start time	H H : M M

SECTION G — Anthropometry

	Measurement type	Unit	Recorded measurements		
1	**Weight** (all participants)	kg	1 _ _ _ , _	2 _ _ _ , _	3 _ _ _ , _
			4 _ _ _ , _	5 _ _ _ , _	**Unable to obtain a measurement**
2	**Height** (all participants)	cm	1 _ _ _ , _	2 _ _ _ , _	3 _ _ _ , _
			4 _ _ _ , _	5 _ _ _ , _	**Unable to obtain a measurement**
3	**Mid upper arm circumference** (3 months and older)	cm	1 _ _ _ , _	2 _ _ _ , _	3 _ _ _ , _
			4 _ _ _ , _	5 _ _ _ , _	**Unable to obtain a measurement**
4	**Triceps Skinfold** (3 months and older)	cm	1 _ _ _ , _	2 _ _ _ , _	3 _ _ _ , _
			4 _ _ _ , _	5 _ _ _ , _	**Unable to obtain a measurement**
5	**Sub scapular Skinfold** (3 months and older)	cm	1 _ _ _ , _	2 _ _ _ , _	3 _ _ _ , _
			4 _ _ _ , _	5 _ _ _ , _	**Unable to obtain a measurement**
6	**Biceps Skinfold** (18 years and older)	cm	1 _ _ _ , _	2 _ _ _ , _	3 _ _ _ , _
			4 _ _ _ , _	5 _ _ _ , _	**Unable to obtain a measurement**
7	**Supra-iliac Skinfold** (18 years and older)	cm	1 _ _ _ , _	2 _ _ _ , _	3 _ _ _ , _
			4 _ _ _ , _	5 _ _ _ , _	**Unable to obtain a measurement**
8	**Waist circumference** (3 months and older)	cm	1 _ _ _ , _	2 _ _ _ , _	3 _ _ _ , _
			4 _ _ _ , _	5 _ _ _ , _	**Unable to obtain a measurement**
9	**Hip circumference** (3 months and older)	cm	1 _ _ _ , _	2 _ _ _ , _	3 _ _ _ , _
			4 _ _ _ , _	5 _ _ _ , _	**Unable to obtain a measurement**

Clinic Assistant	End time	H H : M M

INSTRUCTIONS	Sections H – J to be completed by the Nurse

Nurse	Start time	H	H	:	M	M

SECTION H — Spirometry (18 yrs and older)

Note: *Participants can be excluded from this test on clinical grounds (eg. asthma)*		
1	Was the spirometry test performed?	Yes .... 1<br>No 2
2	If the test was not done, specify the reason	Asthma .... 1<br>Other (specify) .... 2<br>............

Please record the following:

Temperature (°C)	__ __ . __	*Humidity(%)*	__ __	*Pressure (hPa)*	__ __ __ __

	Measurement	Units	Recorded measurements			Final Measurement
3	Forced vital capacity (FVC)	L	1 __ __ , __ __ __	2 __ __ , __ __ __	3 __ __ , __ __ __	4 __ __ , __ __ __
4	Forced expiratory volume (FEV-1)	L	1 __ __ , __ __ __	2 __ __ , __ __ __	3 __ __ , __ __ __	4 __ __ , __ __ __
5	FEV-1/FVC ratio	L	1 __ __ , __ __ __	2 __ __ , __ __ __	3 __ __ , __ __ __	4 __ __ , __ __ __
6	Forced expiratory flow 25-75% (FEF 25-75)	L	1 __ __ , __ __ __	2 __ __ , __ __ __	3 __ __ , __ __ __	4 __ __ , __ __ __

SECTION I — Bioelectrical impedance (18 yrs and older)

Note: *Participants can be excluded from this test on clinical grounds*<br>***Note:*** *Variations in body composition may occur in patients pre-menstrually, during menstruation, with renal failure, on some prescription medications (particularly Diuretics) and other conditions causing water retention or water loss*		
1	Was the bioelectrical impedance test performed?	Yes .... 1<br>No 2
2	If the test was not done, specify the reason	Pace maker .... 1<br>Artificial limbs/joints .... 2<br>Hearing aid .... 3<br>Diuretics .... 4<br>Alcohol consumed .... 5<br>Full bladder 6

	Measurement		Recorded measurements			Final Measurement
3	Z-ohm value	ohms	1 __ __ __ __ , __	2 __ __ __ __ , __	3 __ __ __ __ , __	4 __ __ __ __ , __

SECTION J	Blood collection		
**No.**	**Biomarkers**	**Respondent category (age)**	**Specimen Collected**
1	Full blood count	2 years and older	Yes....1<br>No ....2<br>Missing value – failure to collect a specimen 3
2	Cholesterol (total)	6 years and older	Yes....1<br>No ....2<br>Missing value – failure to collect a specimen 3
3	HDL	6 years and older	Yes....1<br>No ....2<br>Missing value – failure to collect a specimen 3
4	LDL	6 years and older	Yes....1<br>No ....2<br>Missing value – failure to collect a specimen 3
5	Triglycerides	6 years and older	Yes....1<br>No ....2<br>Missing value – failure to collect a specimen 3
6	Glucose (plasma)	6 years and older	Yes....1<br>No ....2<br>Missing value – failure to collect a specimen 3
7	C-reactive protein	6 years and older	Yes....1<br>No ....2<br>Missing value – failure to collect a specimen 3
8	Cotinine	10 years and older	Yes....1<br>No ....2<br>Missing value – failure to collect a specimen 3
9	Zinc status	0 – 5 years, and<br>women 16 – 35 years	Yes....1<br>No ....2<br>Missing value – failure to collect a specimen 3
10	Vitamin A status	0 – 5 years, and<br>women 16 – 35 years	Yes....1<br>No ....2<br>Missing value – failure to collect a specimen 3
11	Iron status	0 – 5 years, and<br>women 16 – 35 years	Yes....1<br>No ....2<br>Missing value – failure to collect a specimen 3

**Nurse**	**End time**	H	H	:	M	M
